# Identification of miRNAs and their targets from *Brassica napus* by high-throughput sequencing and degradome analysis

**DOI:** 10.1186/1471-2164-13-421

**Published:** 2012-08-24

**Authors:** Miao Y Xu, Yun Dong, Qiu X Zhang, Lan Zhang, Yan Z Luo, Jie Sun, Yun L Fan, Lei Wang

**Affiliations:** 1 College of Agriculture/Key Laboratory of Oasis Ecology Agriculture of BINTUAN, Shihezi University, Shihezi, 832003, China; 2 Biotechnology Research Institute, National Key Facility of Crop Gene Resources and Genetic Improvement, Chinese Academy of Agricultural Sciences, Beijing, 100081, China; 3 Crops institute, Gansu Academy of Agricultural Sciences, Lanzhou, 730070, China

## Abstract

**Background:**

MicroRNAs (miRNAs) are endogenous regulators of a broad range of physiological processes and act by either degrading mRNA or blocking its translation. Oilseed rape (*Brassica napus*) is one of the most important crops in China, Europe and other Asian countries with publicly available expressed sequence tags (ESTs) and genomic survey sequence (GSS) databases, but little is known about its miRNAs and their targets. To date, only 46 miRNAs have been identified in *B. napus.*

**Results:**

Forty-one conserved and 62 brassica-specific candidate *B. napus* miRNAs, including 20 miRNA* sequences, were identified using Solexa sequencing technology. Furthermore, 33 non-redundant mRNA targets of conserved brassica miRNAs and 19 new non-redundant mRNA targets of novel brassica-specific miRNAs were identified by genome-scale sequencing of mRNA degradome.

**Conclusions:**

This study describes large scale cloning and characterization of *B. napus* miRNAs and their potential targets, providing the foundation for further characterization of miRNA function in the regulation of diverse physiological processes in *B. napus*.

## Background

Endogenous small RNAs (sRNAs) are known to be important regulators of gene expression at the transcriptional and post-transcriptional levels. They fall into a number of different classes in plants: transacting siRNAs (tasiRNAs), heterochromatin-associated siRNAs, natural antisense siRNAs (nat-siRNAs) and miRNAs [1]. These classes of non-coding RNAs are distinguished by their biogenesis pathways and the types of genomic loci from which they arise [2].

miRNAs are non-coding RNAs of approximately 21 nucleotides that have been identified as important regulators of gene expression in both animals and plants [2-5]. Plant miRNAs are generated from hairpin-structured non-coding transcripts by DCL1(DICER-LIKE 1), which cleaves a short (21 bp) duplex from the stem region [6]. The duplex is incorporated into an AGO1 complex and the miRNA* strand is subsequently degraded. The mature miRNA strand guides the AGO1 complex (RNA-induced silencing complex, RISC) to protein-coding RNAs, which are cleaved by AGO1 at a specific position (opposite to the 10th and 11th nucleotides of the miRNA) [7]. Recent findings have shown that the inhibition of gene expression via translational arrest by the miRNA-guided AGO complex is more common in plants than was previously believed [8]. The mature miRNAs function within large complexes to negatively regulate specific target mRNAs. Plant miRNAs generally interact with their targets through perfect or near-perfect complementarity and direct mRNA target degradation [9,10]. Due to their evolutionary conservation, miRNAs have been found to exist in both plants [9,11] and animals [12-14]. Conserved miRNA molecules can also be found in ferns, mosses and fungi [15,16].

In plants, miRNAs not only post-transcriptionally regulate their own targets but also interact with each other in regulatory networks to affect many aspects of development, such as developmental timing [17-21], senescence [22-24], leaf morphogenesis [25-31], reproductive development [32-35], and modulation of root architecture [36-42]. miRNAs are also reported to be involved in plant responses to biotic and abiotic stresses [31]. To date an increasing number of miRNAs have been identified and deposited in miRBase V17.0 (http://www.mirbase.org/). Among these miRNAs, there are 19,724 plant miRNAs and miRNAs*, from a total of 153 species. The species with the fastest growing number of miRNAs is *Brachypodium distachyon*, with 120 miRNAs being recently added. Initially, miRNAs were identified by the traditional Sanger sequencing method, which used for relatively small-size cDNA libraries of plant sRNAs from *Arabidopsis*, rice and poplar (*Populus* spp.). Comparison of miRNAs from these species led to the conclusion that plant miRNAs are highly conserved [16]. This was supported by observations that even ferns shared common miRNAs with flowering plants [43]. However, it was also noticed that a small number of miRNAs were not present in the genomes of some species, suggesting that they have evolved more recently [25]. As non-conserved miRNAs are often expressed at a lower level than conserved miRNAs, many non-conserved miRNAs were not found in small-scale sequencing projects. However, high-throughput sequencing technologies have allowed the identification of many non-conserved miRNAs in several species [44-47]. To date, hundreds of miRNAs have been isolated by direct cloning or by deep sequencing in higher plants [48]. Elucidating the function of these tiny molecules requires efficient approaches to identify their targets. Originally, plant miRNA targets have been studied via computational prediction, which is based on either perfect or near -perfect sequence complementarity between miRNA and the target mRNA or sequence conservation among different species [10]. However, target prediction is very challenging, especially when a high level of mismatches exists in miRNA:target pairing [49]. Recently, a new method called degradome sequencing, which combines high-throughput RNA sequencing with bioinformatic tools, has been successfully established to screen for miRNA targets in *Arabidopsis*[50-52]. Using degradome sequencing, many of the previously validated and predicted targets of miRNAs and tasiRNAs were verified [50,51,53,54], indicating that it is an efficient strategy to identify sRNA targets on a large scale in plants.

Rape (*Brassica napus*) is one of the most important oil crops, and also is one of the major economic crops. However, unlike *Arabidopsis* and other plants, much less is known about its miRNA classification and miRNA targets, especially the roles of miRNAs in the developmental process of *Brassica napus*. Currently, miRBase lists 46 miRNAs forming 17 miRNA families in *Brassica napus*. The exploration of sRNA-based regulatory networks in *Brassica napus* is an important step towards our better understanding of sRNA-based genic regulation. Here, we describe the high-throughput sequencing analysis of sRNAs from a cultivated variety of *B. napus*, cv Westar, using the Illumina Solexa platform.

The sRNAs library was prepared for Solexa sequencing from greenhouse cultivated rape plants, and produced more than 2 million unique sequences. The most abundant classes were represented by 21 and 24 nt-long sRNAs. Forty-one conserved *B. napus* miRNAs and 62 candidate novel *B. napus*-specific miRNAs were firstly identified. Twelve conserved miRNAs and 10 *B. napus*-specific candidates were further verified by real-time RT-PCR. To identify miRNA targets, a degradome sequencing approach was used, which globally identifies the remnants of sRNA-directed target cleavage by sequencing the 5^′^ ends of uncapped RNAs [50,51]. We identified a total of 33 non-redundant target ESTs for 25 conserved miRNAs, and 19 non-redundant target ESTs for 17 *B. napus*-specific miRNAs. Approximately 70% of the identified targets for conserved miRNAs were transcriptional factors.

## Results and discussion

### Sequencing *B. napus* miRNAs using Solexa technology

We used Solexa technology to deeply sequence *B. napus* sRNAs. Total RNAs from different *B. napus* tissues were pooled and submitted for small RNA sequencing. A total of 13,020,106 reads were generated from the sequencing machine. After removing adaptor sequences, filtering out low quality tags and cleaning up sequences derived from adaptor-adaptor ligation, 2,149,116 unique sequences were obtained. Among these unique sequences, 73,931 (3.44%) were found to be similar to known miRNAs (Table 1).

**Table 1:** **Statistics of sRNA sequences from *B. napus***

	**Redundant**		**Non-redundant**	
	**Number of counts**	**% of total**	**Number of unique sequence**	**% of total**
Raw reads	13020106	\	2149116	\
Adaptor removed	30673	0.24	25713	1.2
Junk Filter ^a^	5182	0.04	2362	0.11
Length filter	1379794	10.6	646357	30.08
Simple sequence filter ^b^	69175	0.53	11859	0.55
Copy number <3	1219472	9.37	1130389	52.6
Hit mRNA, RFam, Repbase	9877816	75.87	57637	2.68
Mappable	437994	3.36	30400	1.41

^a^ the sequences are filtered out if they contain 3 Ns (N is undetermined nucleotide) and only A and C without G and T, vice versa; ^b^ the simple sequences are filtered out if they contain 2 Ns (N is undetermined nucleotide), 7 consecutive As, 8 consecutive Cs, 6 consecutive Gs or 7 consecutive Ts and 10 repeats of any dimer, 6 repeats of any trimer, or 5 repeats of any tetramer. These numbers were from the statistics of miRbase ver16.

**Figure 1:**
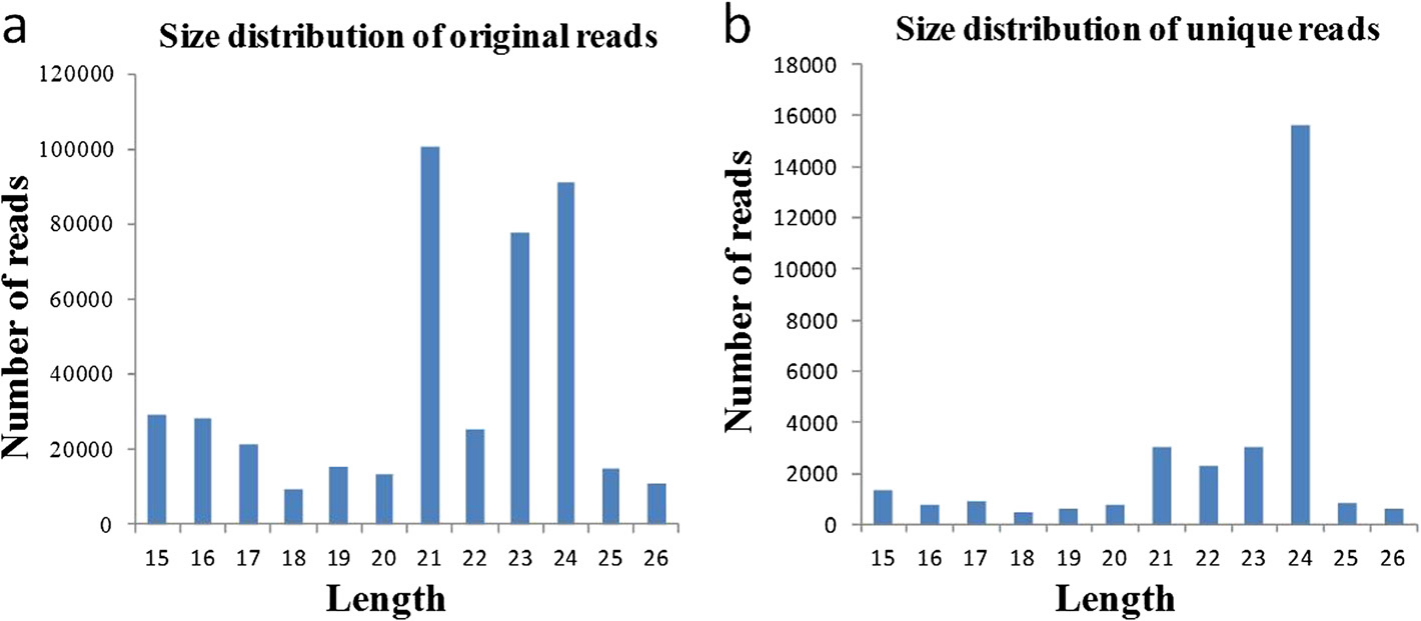
Size distribution of sequenced small RNAs.

SRNAs with known function were commonly 20–24 nt in size [53]; therefore, we analyzed the size distribution patterns of the original and unique reads (Figure 1). The majority of sRNAs were 21 nt in size, followed by 24 nt and 23 nt (Figure 1a), which is consistent with the typical size distribution of sRNAs from other plants. The 21 nt class showed the highest redundancy, whereas the 24 nt class showed lower redundancy (Figure 1a and b).

### Identification of conserved *B. napus* miRNAs

Conserved families of miRNAs are found in many plant species and have important functions in plant development and responses to stresses [55]. In this study, to identity the conserved miRNAs in *B. napus*, our dataset was mapped onto the the genome and ESTs of *B. napus*, *B. rapa* and *B. oleracea,* allowing one or two mismatches between sequences. all retained sequences were compared to known miRNAs from miRBase 17.0 (http://www.mirbase.org/), and secondary structures of these matched miRNAs were predicted. Based on genome mapping and the miRbase results and hairpin prediction, a total of 55conserved miRNAs derived from *B. napus* were identified, including 41 miRNAs and miRNAs* (22 families) were firstly identified together with 14 already in miRbase (Additional file 1: Figure S1, Additional file 2: Table S2). 41conserved miRNAs and miRNAs* were potentially generated from 26 non-redundant ESTs and 3 genomic survey sequence (GSS) loci (Table 2; Additional file 3: Table S3). The precursors of four miRNAs named Bna-miR166f, Bna-miR824*, Bna–miR1140b and Bna–miR1140b* were matched in the genome of *B. rapa* (Additional file 3: Table S3).

**Table 2:** **Conserved miRNAs in *B. napus***

**miRNA**	**miR sequence (5'→3')**	**miR length (nt)**	**Reads**	**Precursor from EST**	**Mature miRNA position**		**Stem-loop position**	
					**miR start**	**miR end**	**Precursor start**	**Precursor end**
Bna--miR159*	GGGCTCCTTATAGTTCAAACG	21	79	EX039355	189	209	186	368
Bna--miR159b	TTTGGATTGAAGGGAGCTCTT	21	66	EV097138	224	244	216	408
Bna-miR160a	TGCCTGGCTCCCTGTATGCCA	21	4447	ES904429	153	173	149	237
Bna-miR160a*	GCGTACAGAGTAGTCAAGCATA	22	7	ES904429	214	235	149	237
Bna-miR161*	GCAAGTCGACTTTGGCTCTG	20	97	BZ512955	431	451	329	463
Bna-miR162a	TCGATAAACCTCTGCATCCAG	21	193	DY025212	495	515	420	523
Bna-miR162b	TCGATAAACCTCTGCATCCAG	21		EV208491	295	315	204	329
Bna-miR162b*	GGAGGCAGCGGTTCATCGATC	21	32	EV208491	222	242	212	323
Bna-miR165a	TCGGACCAGGCTTCATCCCCC	21	2205	FP063045	53	73	34	160
Bna--miR166a*	GGAATGTTGTTTGGCTCGAAG	21	29	DX911364	806	826	697	831
Bna-miR166e	GGAATGTTGTCTGGCTCGAGG	21	328	CU967744	48	68	39	185
Bna-miR167d	TGAAGCTGCCAGCATGATCTA	21	6110	CT022223	721	741	635	758
Bna-miR167e	TGAAGCTGCCAGCATGATCTA	21		AC189327	249	269	153	286
Bna-miR167e*	GATCATGTTCGTAGTTTCACC	21	47	AC189327	169	189	153	286
Bna-miR167f	TGAAGCTGCCAGCATGATCTT	21	160	ES910254	45	65	43	134
Bna-miR168b	TTCGCTTGGTGCAGGTCGGGA	21	14	DU984956	357	377	258	396
Bna-miR169n	GCAAGTTGACTTTGGCTCTGT	21	1463	CU944678	404	424	354	528
Bna-miR169n*	TGAGCCAAAGATGACTTGCCG	21	11	CU944678	459	479	356	530
Bna-miR171a*	AGATATTAGTGCGGTTCAATC	21	7	DX044654	128	148	119	219
				DU980843	223	243	159	246
Bna--miR171f*	TATTGGCCTGGTTCACTCAGA	21	34	DU106747	666	686	647	756
Bna-miR172a	GGAATCTTGATGATGCTGCAT	21	95	EV092015	731	751	637	769
Bna-miR172a*	GTGGCATCATCAAGATTCACA	21	3	EV092015	654	674	629	777
Bna-miR172b	AGAATCTTGATGATGCTGCAT	21	223	CU946172	157	177	69	189
Bna-miR319a	GAGCTTTCTTCGGTCCACTC	20	105	ES908144	308	327	301	477
Bna-miR319b-1	ATCTGCCGACTCATCCATCCA	21	11	CN829704	153	173	76	312
Bna-miR319b-2	GAGATTCTTTCAGTCCAGTCA	21	3	CN829704	103	123	74	310
Bna-miR390d	AAGCTCAGGAGGGATAGCGCC	21	1157	EE544982	541	561	471	581
Bna-miR390d*	CGCTGTCCATCCTGAGTTTCA	21	348	EE544982	441	461	421	531
Bna -miR393*	ATCATGCGATCTCTTCGGATT	21	30	DU101699	224	242	116	242
Bna-miR396	AATAAAGCTGTGGGAAGATAC	21	24	DU106522	54	74	35	216
Bna-miR398	TGTGTTCTCAGGTCACCCCTG	21	66	EE426846	89	109	1	123
Bna-miR399d	TGCCAAAGGAGATTTGCCCGG	21	71	CX190537	106	126	9	146
Bna-miR399f	TGCCAAAGGAGAGTTGCCCTG	21	62	EE556998	584	604	475	635
Bna-miR400	TATGAGAGTATTATAAGTCAC	21	25	CX189066	239	259	212	346
Bna-miR408a	ACAGGGAACAAGCAGAGCATG	21	305	ES903146	59	79	49	157
Bna-miR408a*	ATGCACTGCCTCTTCCCTGGC	21	141	ES903146	128	148	49	157
Bna -miR2111c	TAATCTGCATCCTGAGGTTTA	21	35	BH986382	394	414	375	493
				DX056967	280	300	176	326

The read number of the conserved miRNAs was highly variable, indicating different expression levels of these miRNAs. Among them, Bna-miR159, Bna-miR166a, Bna-miR164, Bna-miR171f and Bna-miR168 had relatively high number of reads, indicating that these miRNAs are likely to be expressed at a higher level, whereas Bna-miR169 family members had a low number of reads, and are, therefore, likely to be expressed at a lower level (Additional file 4: Table S1). The relative expression level of a few known miRNA families, such as miR159, miR167, miR160, miR165 and miR390, were similar to that of *Arabidopsis*[44] (Table 2).

### Brassica-specific miRNAs

A distinct feature of miRNAs is the ability of their pre-miRNA sequences to adopt the canonical stem-loop hairpin structure. After removal of conserved miRNAs, the rest sRNA reads were predicted for each mapped locus for potential stem-loop structures. From this analysis, we identified 62 miRNA and miRNA*candidates (47 families) that were potentially generated from 62 EST or GSS loci (Additional file 5: Figure S2, Table 3).

**Table 3:** Candidate new brassica-specific miRNAs

**miRNA**	**miR sequence (5'→3')**	**miR length (nt)**	**Reads**	**Precursor from EST**	**Mature miRNA position**		**Stem-loop position**	
					**miR start**	**miR end**	**Precursor start**	**Precursor end**
Bna-miRC1	CCATACTAAATCTGGATCATTT	22	115	CU943501	519	540	519	634
Bna-miRC2	ATAAATCCCAAGCATCATCCA	21	1011	EV202910	179	199	173	261
Bna-miRC3	TGGGATTGGCTTTGGGCTTTTC	22	12	CU940792	108	129	82	281
Bna-miRC4	TTTCAGTCGTCATAGGTTAGT	21	11	GT084890	55	75	51	158
Bna-miRC5-1	TGTGTTGTGATGATAATCCGA	21	306	CU971106	285	305	132	350
Bna-miRC5-1*	AATCGGATTATCATCACAACA	21	7	CU971106	93	113	29	291
Bna-miRC5-2	TCAACCAAATACACATTGTGG	21	4	CU971106	52	72	35	306
Bna-miRC5-3	TTATCATCACAACACTAGATC	21	536	CU971106	76	96	19	281
Bna-miRC5-3*	TCTTGTGTTGTGATGATAATC	21	216	CU971106	288	308	136	349
Bna-miRC5-4	TGATAATCCGACTTCTATGAC	21	29	CU971106	272	292	122	356
Bna-miRC5-5	TTGGTTTGGATCTTGGAAATC	21	8	CU971106	123	143	42	304
Bna-miRC5-6	TCGGATTATCATCACAACACT	21	182	CU971106	89	109	27	289
Bna-miRC6	ATAGATCCTTCTGATGACGCA	21	16	DU099814	306	326	254	327
Bna-miRC7	CAAATCCTGTCATCCCTACCA	21	89	GT079632	102	122	102	229
Bna-miRC8	CAGGAGAGATTGTTGGATCCA	21	3	CU931338	337	357	337	443
Bna-miRC9	TGCCTGGCTCCCTGTATACCA	21	118	EV193539	387	407	380	474
Bna-miRC10	TCAATGTTGGCTCAATTATGT	21	12	CU934632	731	751	666	751
Bna-miRC11	GGCGAGTCACCGGTGTCGGTC	21	6	FP328714	415	435	406	534
Bna-miRC12	GGGTCGATATGAGAACACATG	21	15	EE426846	15	35	1	123
Bna-miRC13	ACCCTGTTGAGCTTGTCTCTA	21	3	CU980942	490	510	449	524
Bna-miRC14	CAGCTGGACGACTTAGTAGAC	21	7	CU943399	123	143	103	229
Bna-miRC15-1	ACATTGGACTACATATATTAC	21	8	ES901619	392	412	299	430
Bna-miRC15-2	TCAATACATTGGACTACATAT	21	9	ES901619	387	407	299	430
Bna-miRC16	GTTTTGAGAGATTGGGAAGCT	21	3	EV146378	77	97	58	216
Bna-miRC17a-1	TTTCCAAATGTAGACAAAGCA	21	7132	ES913560	96	116	31	137
Bna-miRC17a-1*	CTTTGTCTATCGTTTGGAAAAG	22	782	ES913560	53	74	31	137
Bna-miRC18	TCGCGATCTTAGATCCTCTAA	21	41	EV179238	441	461	288	564
Bna-miRC19	CGAGTTGGTCGGGAAAGACGG	21	12	DU102764	104	124	35	128
Bna-miRC20	CTCTCGTGGAGCGTCTCGAGG	21	3	EV192419	700	720	567	746
Bna-miRC21	GGAGGCAGCGGTTGATCGATC	21	7	DY025212	429	449	420	523
Bna-miRC22a-1	CAAGTAGACGACTTTCCAGAC	21	10	CU945922	359	379	298	403
Bna-miRC22a-2	CGTGGTCGTCCAAGTAGACGA	21	13	CU945922	363	383	292	397
Bna-miRC22a-3	TTGGACGACTTTGTAGACGAC	21	9	CU945922	303	323	297	402
Bna-miRC23a-1	TCAGAACCAAACCCAGAACAAG	22	54	CU958057	25	46	3	241
Bna-miRC23a-2	TTACAGAACAGCAACAAGCTGT	22	150	CU958057	47	68	7	238
Bna-miRC23a-3	TATCTACTGCTTATGCCACCA	21	65	CU958057	54	74	1	215
Bna-miRC23a-3*	GATGCATAACCACTAGATACG	21	8	CU958057	140	160	1	215
Bna-miRC24	TTAGGATTGAGATCTTAGCGA	21	7	EV176533	225	245	214	395
Bna-miRC25	TTGGACTGAAGGGAACTCCCT	21	23719	FP023833	319	339	168	343
Bna-miRC25*	AGAGTTTCCTTAAGTCCATTC	21	34	FP023833	173	193	167	343
Bna-miRC26	TGAGCCAAAGATGACTTGTCG	21	45	BZ021311	68	88	66	323
Bna-miRC27	TAAGATGATGGAACACTGGCC	21	25	EE438385	18	38	14	279
Bna-miRC28	ATGGATCCGCCGGATAAGGAT	21	6	CU965419	466	486	350	511
Bna-miRC29	TTGAGGTTTTGAGGACTGGCC	21	6	EV093069	644	664	564	668
Bna-miRC30	TCCTGGACGACTTTCAAGTAAG	22	9	CZ888137	161	182	20	250
Bna-miRC31	AGATCATCCTGCGGCTTCATT	21	26	EV134163	290	310	233	335
Bna-miRC32	GCAAGTTGACTTTGGCTCCGT	21	51	BZ021311	52	72	13	184
Bna-miRC33	TTTTGCCTACTCCTCCCATACC	22	268	CU981257	103	124	95	223
Bna-miRC34	ATCCTCGGGACACAGATTACC	21	113	EV076017	357	377	339	459
Bna-miRC35	ATGGTGTAGGTACTGAGCAGA	21	13	EV194620	298	318	294	400
Bna-miRC36	CGTCCGGGGAAAGCAAAGTCG	21	11	EV088144	141	161	64	186
Bna-miRC37	TGATTTATCCAAGGGTTCAGG	21	31	DU101557	509	529	367	608
Bna-miRC38	CAAGTAGACTACTTTCCAGACG	22	9	GT084321	52	73	1	92
Bna-miRC39	TAAGATGATGGGACGTTGGATC	22	11	DY002174	42	63	40	306
Bna-miRC40	CGCTCACAGCATCTGAACTCT	21	21	CD842549	99	119	78	243
Bna-miRC41	TTTTGGAGAAGGCTGTAGGCA	21	13	DU109430	791	811	780	890
Bna-miRC42	TTCCCCGGACGACTTTAAATT	21	15	EV088144	90	110	3	125
Bna-miRC43	TGTGAATGATGCGGGAGATGT	21	15	CN829704	219	239	69	315
Bna-miRC44	TTGGCCACAACGGATTTAACA	21	9	EV006438	66	86	66	141
Bna-miRC45	TTTCATCTTAGAGAATGTTGTC	22	42	EV178795	578	599	478	617
Bna-miRC46	ACTTGTCTCACTCATCAGTT	20	7	EV063926	5	24	3	215
Bna-miRC47	CAAATGTAGACAAAGCAAAAC	21	4	ES913560	100	120	31	137

**Figure 2:**
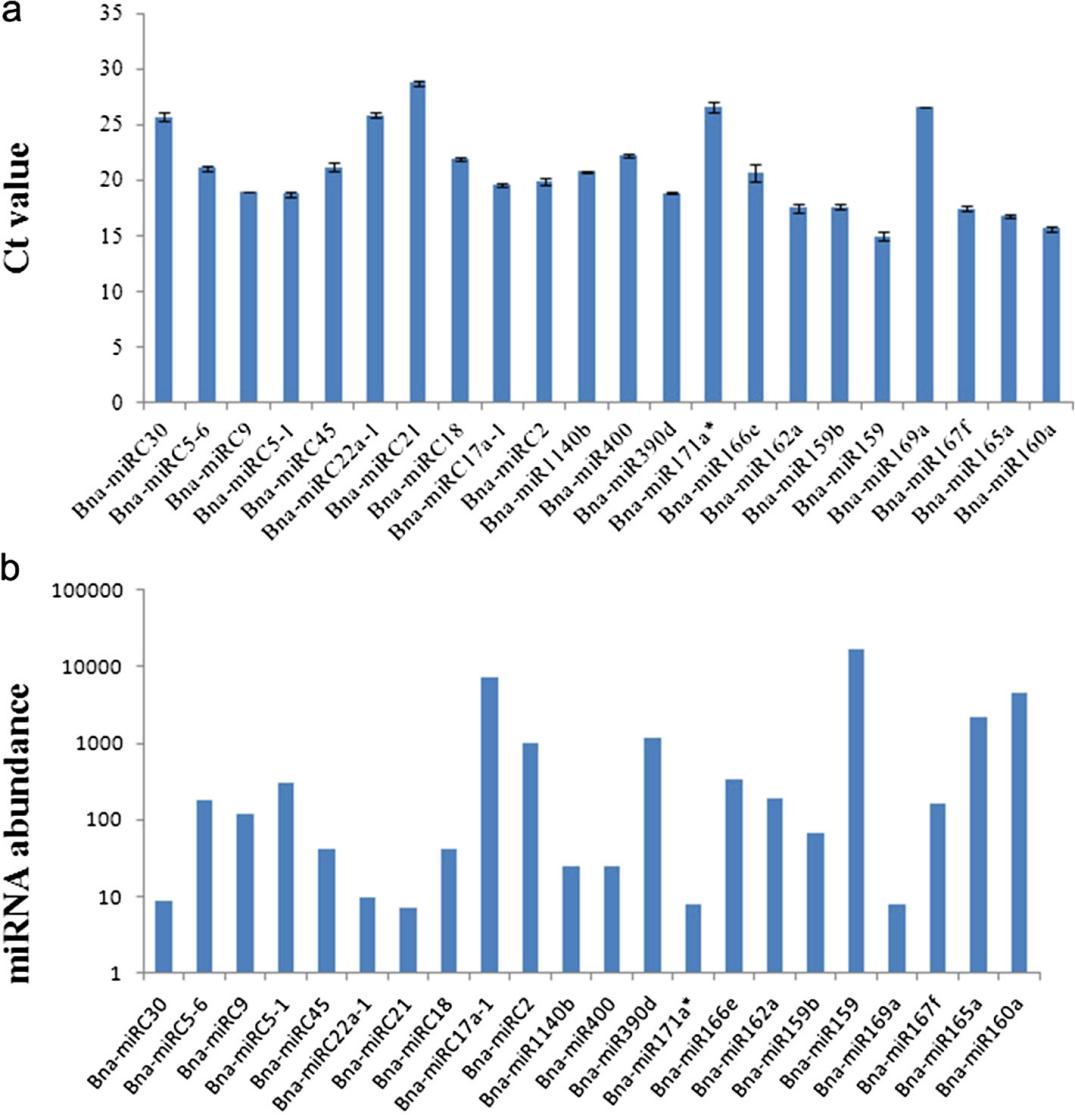
**Expression levels of Bna-miRNAs by two methods.** (**a**) Profile of qRT-PCR Ct values for Bna-miRNAs; (**b**) Profile of sequencing frequencies for Bna-miRNAs.

Generally, new species-specific miRNAs are considered to be young miRNAs that have evolved recently, and are often expressed at a lower level than conserved miRNAs, as was reported for *Arabidopsis* and wheat [44,46,56]. This observation is also true for many of the new *B. napus* miRNAs identified here. However, few new miRNAs were expressed at a high level, which was opposite with this observation (Figure 2). In some cases we observed considerable inconsistency between the level of miRNAs identified by Solexa sequencing and quantitative RT-PCR (qRT-PCR) analysis, however, though we do not know the explanation for these differences. It is possible that the primers used for stem-loop real-time reactions can bind miRNA species with a few mismatches that were not considered by the bioinformatic analysis.

### Stem-loop qRT-PCR validation and measurement of *B. napus* miRNAs

To verify the existence of the newly identified rape miRNA candidates, the same RNA preparation used in the Solexa sequencing was subjected to stem-loop qRT-PCR [57]. Finally, Twelve conserved miRNAs and 10 brassica-specific candidates, which were randomly selected, could be readily detected by qRT-PCR (Figure 2a), suggesting that miRNAs are bona fide miRNAs. Most results of qRT-PCR analysis agreed with the sequencing data, as in the cases of Bna-miR159, Bna-miR159b, Bna-miR160a, Bna-miR165a, Bna-miR166e, Bna-miR167f, Bna-miR169a, Bna-miR171a*, Bna-miR390d, Bna-miR400, Bna-miR1140b, Bna-miRC2, Bna-miRC5-1, Bna-miRC5-6, Bna-miRC17a-1, Bna-miRC18, Bna-miRC21, Bna-miRC22a-1, Bna-miRC30 and Bna-miRC45. In some cases, however, a discrepancy was also observed between the qRT-PCR and sequencing data (Bna-miR162a and Bna-miRC9; Figure 2a, b; Table 2, 3). The results suggested that Solexa sequencing was capable of successfully discovering candidate novel miRNAs from this species with high accuracy and efficiency.

### Targets of known *B. napus* miRNAs

**Figure 3:**
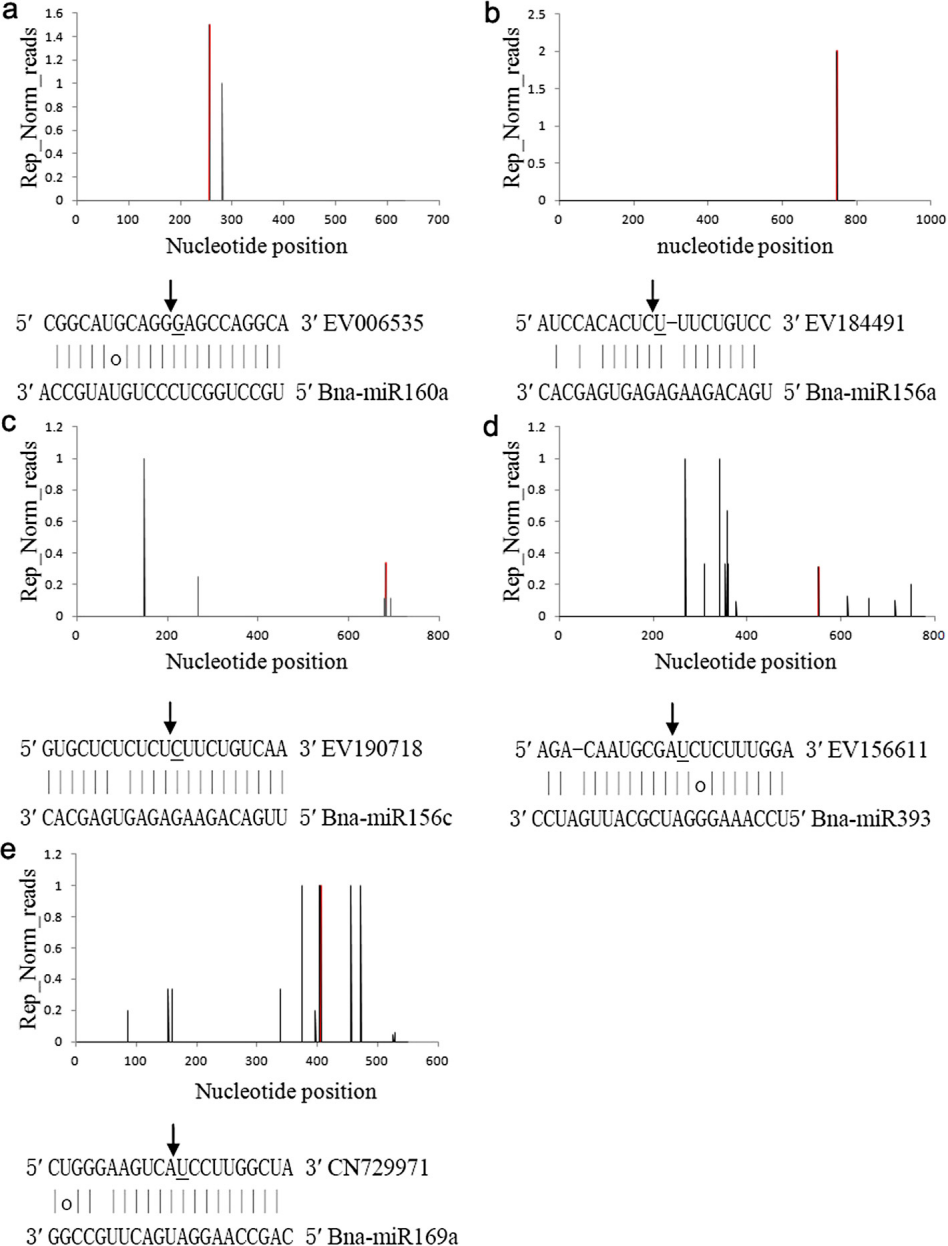
**Confirmed microRNA (miRNA) targets using degradome sequencing are presented in the form of target plots (t-plots).** We used normalized numbers in plotting the cleavages on target mRNAs, which were referred to as ‘target plots’ (t-plots) by German et al. [51]. Signature abundance throughout the length of the indicated transcripts is shown. Representative t-plots for class 0 (**a**), class I (**b**), class II (**c**), class III (**d**), and class VI (**e**) categories are shown. Arrows indicate signatures consistent with miRNA-directed cleavage. miRNA:mRNA alignments along with the detected cleavage frequencies (normalized numbers) are shown. The frequencies of degradome tags with 5′ends at the indicated positions are shown in black, with the frequency at position 10 of the inset miRNA target alignment highlighted in red. The underlined nucleotide on the target transcript indicates the cleavage site detected in the degradome.

In *B. napus*, many conserved miRNA targets have been predicted previously [58,59], but few miRNA targets were identified experimentally. We therefore employed the recently developed high-throughput experimental approach [50,51,60] allowed us to identify target genes for known miRNAs and candidate new miRNAs identified in this work. The poly-A fraction of a balanced total RNA mix from leaf, petiole, stalk and root tissue was analyzed for the identification of target transcripts of known and new miRNAs. We obtained a total number of 8, 356, 060 reads with an average size of ~20 nt, representing the 5^′^ ends of uncapped, polyadenylated RNAs. After initial processing, 6,999,869 reads were obtained, and could be mapped to mRNAs. Previous studies established that the 5^′^ terminal nucleotide of miRNA-cleaved mRNA fragments would correspond to the nucleotide that is complementary to the 10th nucleotide of the miRNA. Therefore, the cleaved RNA targets should have distinct peaks in the degradome sequence reads at the predicted cleavage site relative to other regions of the transcript [50,51]. CleaveLand pipeline [60] was used to identify cleaved targets for both known and new miRNAs in *B. napus*. The abundance of the sequenced tags was plotted for each transcript, and the results are shown in Figures 3, Additional file 6: Figure S3 and Additional file 7: Figure S4. The cleaved target transcripts were categorized into five classes (categories 0, 1, 2, 3 and 4). There were 31 non-redundant ESTs identified as known miRNAs targets, covering 17 miRNA families (Table 4). Nine target ESTs were classified as category 0 (Figure 3a). Category 0 targets are transcripts where the degradome reads corresponding to the expected miRNA-mediated cleavage site were the most abundant reads matching the transcript and there is only one peak on the transcript with more than one raw read at the position. Transcripts of one target (EV184491, for Bna-miR156a) fall into category 1 (Figure 3b), where the total abundance of degradome sequences at the cleavage position is equal to the maximum on the transcript, and there is more than one raw read at the position and more than one maximum position on the transcript. 3 target ESTs were classified as Category 2 (Figure 3c), where abundance at the cleavage position is less than the maximum but higher than the median for the transcript and more than one raw read at the position. 2 target ESTs were classed as Category 3 (Figure 3d), where abundance at the cleavage position is equal to or less than the median for the transcript and more than one raw read at the position. Among the identified targets the most abundant category was category 4 (18 target ESTs), where there is only one raw read at the cleavage position (Figure 3e). Using these classifications we identified targets for 17 conserved miRNA families out of 25. Many highly conserved miRNAs were identified in *B. napus* (Table 2) did not have detectable sliced targets in the degradome sequencing data (e.g. miR161, miR166, miR168 and miR397). It is possible that the levels of conserved miRNAs (e.g. miR161) or sliced targets are below the detection level in this growth stage, and may be present in other specific stages or tissues that have not yet been analyzed. Alternatively, these miRNAs inhibit target gene expression through translational arrest rather than mRNA cleavage.

**Table 4:** **Targets of conserved *B. napus *miRNAs**

**miRNA**	**Target EST**	**Category**	**Cleavge site**	**Reads mapping to the expected cleavage site**	**Percentage of expected reads to total reads mapped to the full length of EST (%)**	**Target site location**	**Target annotation**
Bna-miR156a	EL625881	4	455	5	19	3^′^UTR	A. *thaliana* SPL3 transcription factor
	EV190718	2	681	3	38	3^′^UTR	A. *thaliana* SPL10 transcription factor
	EV184491	1	747	2	100	3^′^UTR	A. *thaliana* O-fucosyltransferase family protein
Bna-miR156c	EV190718	2	682	3	38	3^′^UTR	A. *thaliana* SPL10 transcription factor
Bna-miR159	EV087133	4	439	2	20	ORF	A. *thaliana* MYB65
	EV223870	4	279	5	25	ORF	metallo-beta-lactamase family protein
	EV136053	4	685	3	21	ORF	pyruvate, phosphate dikinase (PPDK)
Bna-miR160a	EV006535	0	256	2	50	ORF	auxin response factor
Bna-miR164b	ES906443	4	68	3	20	ORF	NAC domain-containing protein
Bna-miR167b	ES962471	4	264	3	25	ORF	Auxin response factor 8
Bna-miR167c	EV208388	4	657	3	27	ORF	*B. rapa* IAA-amino acid hydrolase 3
Bna-miR169a	EE543166	0	247	3	50	3^′^UTR	A. *thaliana* NF-YA3
	CN729971	4	406	3	17	3^′^UTR	A. *thaliana* NF-YA5
Bna-miR169e	ES980547	2	459	5	36	3^′^UTR	A. *thaliana* NF-YA3
	EE543166	0	247	3	50	3′UTR	A. *thaliana* NF-YA3
	CN729971	4	406	4	22	3′UTR	A. *thaliana* NF-YA5
Bna-miR169l	ES980547	2	459	5	36	3′UTR	A. *thaliana* NF-YA3
	ES959135	4	352	3	27	3^′^UTR	*B. napus* clone bncbf-b2
	EE543166	0	247	3	50	3^′^UTR	A. *thaliana* NF-YA3
	CN729971	4	406	4	22	3^′^UTR	A. *thaliana* NF-YA5
Bna-miR171b	ES907976	3	609	3	33	ORF	*B. napus* SCL1
	ES902868	3	673	4	34	ORF	A. *thaliana* SCL6-IV
Bna-miR171f	ES902868	4	676	3	21	ORF	A. *thaliana* SCL6-IV
	ES907976	4	612	4	25	ORF	*B. napus* SCL1
Bna-miR171g	ES902868	4	676	3	23	ORF	A. *thaliana* SCL6-IV
	ES907976	4	612	2	18	ORF	*B. napus* SCL1
Bna-miR172f	FG568924	4	488	2	20	ORF	*B. napus* APETALA2
	EV197066	0	677	6	50	ORF	A. *thaliana* AP2-like protein (At2g28550)
	DY025256	4	609	5	24	ORF	A. *thaliana* AP2-like protein (SMZ)
Bna-miR390a	EV220086	0	433	4	50	ORF	cytochrome P450 family 78, subfamily A
Bna-miR390d	EV220086	0	433	4	50	ORF	cytochrome P450 family 78, subfamily A
Bna-miR393	EL628991	2	246	10	17	ORF	A. *thaliana* auxin signaling F-box 3
	EV176346	0	564	6	50	ORF	A. *thaliana* auxin signaling F-box 3
Bna-miR396a	ES923674	4	571	1	20	ORF	A. *thaliana* bHLH74 transcription factor
Bna-miR397b	ES906654	4	736	3	20	ORF	A. *thaliana* laccase-4 (IRX12)
	EE460611	4	445	5	25	ORF	A. *thaliana* 60S ribosomal protein L15
Bna-miR399	EV157460	4	268	3	23	5^′^UTR	A. lyrata PHO2/UBC24
	CX281881	4	581	5	23	ORF	*B. napus* genes for ITS1, ITS2
Bna-miR408a	EE417826	4	457	2	20	ORF	A. *thaliana* peptide chain release factor 1
Bna-miR408b	EV075738	4	63	2	22	5^′^UTR	A. *thaliana* plantacyanin
Bna-miR824	EV112524	0	319	4	50	ORF	A. *thaliana* MADS-box protein AGL16
miR1140b	EV217683	0	473	3	50	ORF	*T. aestivum* mRNA for glycosyltransferase
	ES912747	0	119	1	50	ORF	A. *thaliana* two-component response regulator ARR8 (RR3)
Bna-miR2111b	EV221566	0	337	3	50	ORF	A. *thaliana* F-box/kelch-repeat protein

Most of the identified targets of the conserved *B. napus* miRNAs belong to diverse gene families of transcription factors, such as SPLs, ARFs, MYBs, NF-Y subunits, NAC-domain proteins, AP2-like factors, SCLs and MADS-box factors (Table 4). Many of these transcription factors are known to regulate diverse aspects of plant growth and development. For example, SPLs and AP2-like factors targeted by miR156 and miR172, respectively, have been shown to play an important role in phase changes (from juvenile to adult and from vegetative to the reproductive phase) in *Arabidopsis*[21]. Another important family of transcription factors is the MADS-box gene family,which is known to play a critical role in determining organ specificity during flower development in *Arabidopsis*[61]. One MADS-box gene (AtAGL16-like protein) was validated as a target for miR824 in *B. napus* (Table 4). MADS-box factors in *B. napus* have also been identified to play important roles in petal identity [62]. Similarly, three SCL6s targeted by miR171 play an important role in the regulation of shoot branch production in *Arabidopsis*[63]. Besides their possible involvement in plant development, miRNA targets identified in this study could also play fundamental roles in biotic and abiotic stress resistance in *B. napus*. NF-YA transcription factor genes were validated as targets of for miR169 family numbers. The AtNF-YA5 transcription factor, whose transcript is a target of miR169, has been implicated in drought stress responses in *Arabidopsis*[64]. Over-expression of a miR169-resistant AtNF-YA5 transgene significantly improves drought resistance by promoting stomatal closure under drought stress [64]. Furthermore, NF-YA factors in *Petunia hybrida* and *Antirrhinum majus* were validated to play important roles in floral organ identity [65]. NF- YA mRNAs were identified as targets of miR169 in *B. napus* (Table 4). In addition, laccases (enzymes involved in cell wall metabolism), plantacyanin-like proteins (involved in reproduction and seed setting) and F-box proteins involved in auxin-stimulated protein degradation (TIR1-like) were among the confirmed targets in *B. napus* (Table 4). Bna-miR1140 is a brassica-specific miRNA identified in our previous work.

### Brassica-specific miRNA targets

Out of the 62 candidate new miRNAs, we only identified targets for only 17 miRNAs from the degradome sequencing data, plus 19 non-redundant target ESTs for candidate new brassica-specific miRNAs (Table 5). The abundance of the sequence tags for candidate brassica-specific miRNA target transcripts was plotted as a function of its position in the target genes (Additional file 7: Figure S4). We found there was no clear correlation between the expression level of the new miRNAs and their ability to target an mRNA for cleavage. We found candidate new miRNAs, such as Bna-miRC8, Bna-miRC13, Bna-miRC16, target mRNAs despite their low abundance and that target mRNAs. Consistent with our observation, no clear inverse correlations between the miRNA abundance and the cleavage frequency of target transcripts in *Arabidopsis*, rice and grapevine have been reported [53,66,67]. The new *B. napus* miRNAs target different genes with a wide variety of predicted functions. For instance, Bna-miRC16 targets chlorophyll a/b-binding protein gene, Bna-miRC20-1 targets photosystem II reaction center W-like protein gene and Bna-miRC21 targets photosystem I subunit XI gene, which are all involved in photosynthesis. Bna-miRC17a-1 targets cinnamyl alcohol dehydrogenase (CAD), which is likely to be involved in pathogen resistance and plant development [68]. Several specific targets, such as PPR-containing protein (required for normal plant development), ferrochelatase (involved in the heme biosynthetic pathway), GF14 omega proteins (potential roles in signaling), FtsH-like protease (an ATP-dependent zinc metalloprotease, related to photo-oxidative damage), glycosyl hydrolase family proteins (involved in plant cell wall architecture), Histone H2A and Histone H2B (involved in compacting DNA strands and chromatin regulation) were found as targets of rape-specific miRNAs in *B. napus*.

**Table 5:** **Targets of candidate novel *B. napus *miRNAs**

**miRNA**	**Target EST**	**Category**	**Cleavage site**	**Reads mapping to the expected cleavage site**	**Percentage of expected reads to total reads mapped to the full length of EST (%)**	**Target site location**	**Target annotation**
Bna-miRC2	EV142354	1	347	6	30	ORF	*A. lyrata* PPR-containing protein
Bna-miRC5-2	EV077017	0	326	4	50	ORF	*A. lyrata* exostosin family protein
Bna-miRC5-5Bna-miRC8	EV154449 FG574835	2 4	296 119	3 2	27 20	ORF 5^′^UTR	A. *thaliana* alpha-tubulin 6 A. *thaliana* uncharacterized protein
Bna-miRC9	EV006535	0	256	2	50	ORF	A. *thaliana* auxin response factor 17
Bna-miRC13	EV022057	1	105	3	30	ORF	A. *thaliana* protein PIR
Bna-miRC15-1	EV191962	0	132	5	50	5^′^UTR	A. *thaliana* ferrochelatase 1
Bna-miRC15-2	EV054423	2	615	3	33	ORF	*A. lyrata* ferrochelatase 1
Bna-miRC16	GR445128	3	416	4	27	ORF	*B.juncea* chlorophyll a/b-binding protein
Bna-miRC17a-1	CD818234	1	647	5	36	ORF	A. *thaliana* cinnamyl alcohol dehydrogenase
Bna-miRC18	GT074945	2	341	12	30	ORF	*B.napus* GF14 omega
Bna-miRC20-1	GR442870	0	578	3	50	ORF	A. *thaliana* histone H2B-like protein
	ES987065	0	39	3	50	ORF	*B.rapa* photosystem II center W-like protein
Bna-miRC21	GT079646	2	65	3	33	5^′^UTR	A. *thaliana* photosystem I subunit XI
Bna-miRC22a-1	EV044066	3	505	4	27	ORF	A. *thaliana* OST3/OST6 family protein
Bna-miRC26	EV077764	0	593	5	50	ORF	*A.thaliana* uncharacterized protein (AT3G51610)
Bna-miRC30	EV025081	2	329	4	36	ORF	Glycosyl hydrolase family protein
	CX189212	3	269	3	27	ORF	Glycosyl hydrolase family protein
Bna-miRC47	ES992448	0	517	6	50	ORF	*A. thaliana* prenylcysteine oxidase (FCLY)

### Verification of miRNA-guided cleavage of target mRNAs in *B. napus*

**Figure 4:**
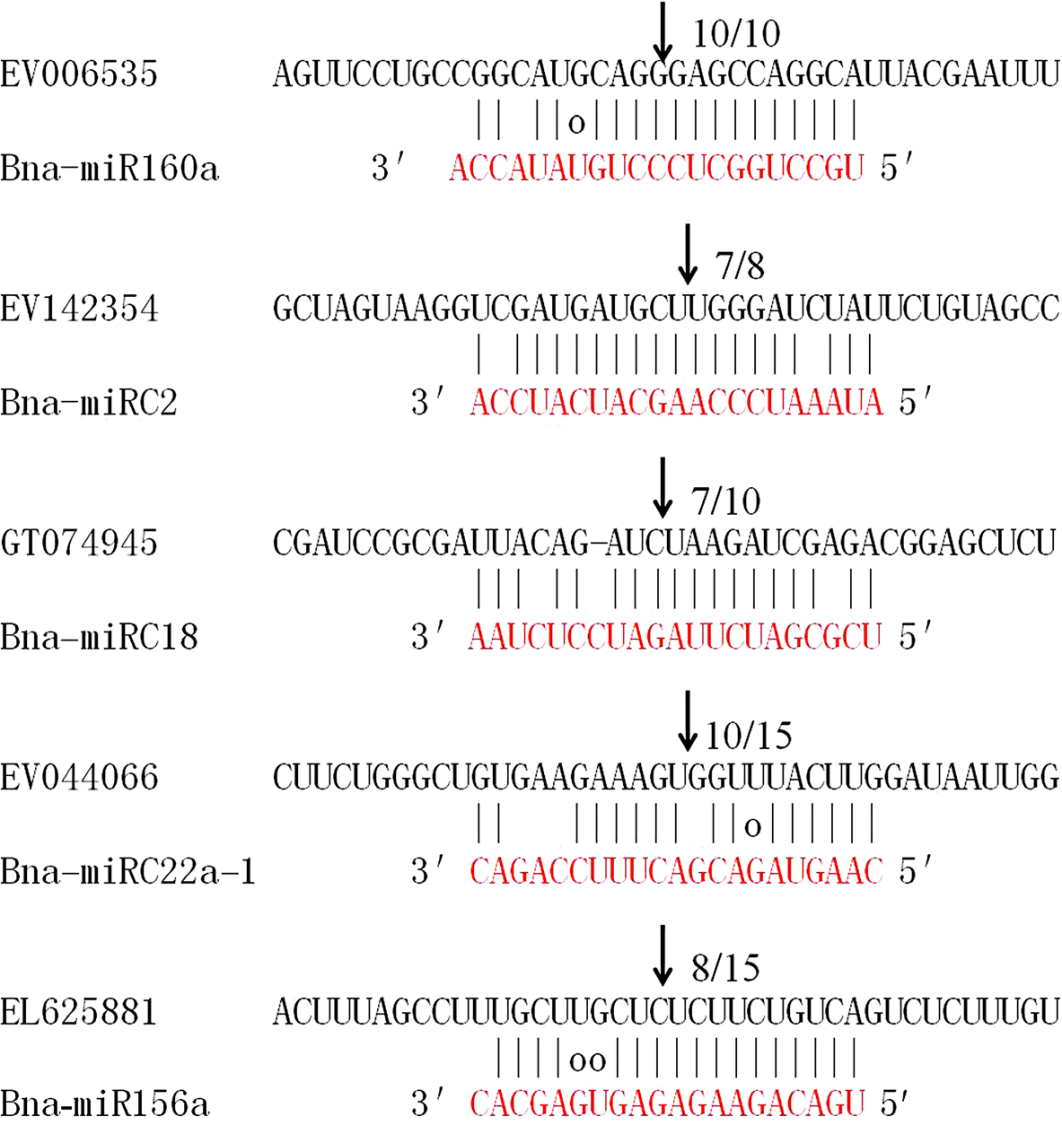
**Mapping of the mRNA cleavage sites by RNA ligase-mediated 5**^′^**RACE.** Each top strand (black) depicts a miRNA complementary site, and each bottom strand depicts the miRNA (red). Watson-Crick pairing (vertical dashes) and G:U wobble pairing (circles) are indicated. The arrows indicate the 5^′^ termini of mRNA fragments isolated from *B. napus*, as identified by cloned 5^′^ RACE products, with the frequency of clones with the predicted cleavage site shown.

To verify the miRNA-guided target cleavage, RLM-RACE experiment was performed to detect cleavage product of 5 predicted Bna-miRNAs (primers were listed in Additional file 8: Table S4). As shown in Figure 4, all five of the Bna-miRNAs guided the target cleavage, often at the tenth nucleotide, or eleventh nucleotide (Figure 4). Thus, all the five predicted targets were found to have specific cleavage sites corresponding to the complementary sequences of miRNA.

## Conclusion

Here, 41 conserved data and 62 brassica-specific candidate miRNAs, including 20 miRNA* sequences were firstly identified. The sequencing results were further confirmed using stem-loop quantitative RT-PCR. The data will be updated to incorporate future miRBase updates. Our approach leads to the identification of several conserved and specific brassica miRNA targets in the available EST and genomic databases. 33 non-redundant mRNA targets for the conserved brassica miRNAs and 19 non-redundant mRNA targets of new brassica-specific miRNAs were identified. Validated miRNA targets in *B. napus* are potentially involved in diverse biological processes, including phase transitions, flowering, hormone signaling, photosynthesis, metabolism and biotic and abiotic stress resistance. Our data will be a useful resource for further investigation of the evolution of small RNA-based regulation in *Brassica napus* and related species. More importantly, this study will serve as a foundation for future research on the functional roles of miRNAs and their target genes in this important oil crop.

## Methods

### Plant materials

The dihaploid *B. napus* line, Westar, was grown in a glasshouse at 22–25°C with a 16 h light/8 h dark photoperiod and light intensity of >8000 lx. Leaves, petiole, stalk, roots and shoot apices from one month-old seedlings were collected and used for RNA extraction. A balanced RNA mix was used for small RNA expression and degradome analysis.

### RNA extraction and preparation of sRNA and degradome cDNA libraries for Solexa sequencing

*B. napus* total RNA from different tissues was extracted using Trizol (Invitrogen). The total RNA balanced mix sample was size-fractionated by 15% denaturing polyacrylamide gel electrophoresis, after which the small RNA fragments of 18–28 nt were isolated from the gel and purified. The small RNA molecules were then ligated to a 5' adaptor and a 3' adaptor sequentially and then converted to cDNA by RT-PCR following the Illumina protocol. The concentration of the sample was adjusted to ~10 nM and a total of 10 μL was used in a sequencing reaction. The purified cDNA library was sequenced on an Illumina GAIIx.

The degradome library was constructed as previously described [51]. Similarly to the short RNA libraries, the degradome cDNA library was sequenced on an Illumina GAIIx.

### Bioinformatic analyses

After masking adaptor sequences and removal of contaminated reads the clean reads were filtered for miRNA prediction with the ACGT101-miR-v3.5 package (LC Sciences, Houston, USA). First, reads that matched rRNA, tRNA, snRNA, snoRNA, repeat sequences, and other ncRNAs deposited in Rfam (http://www.sanger.ac.uk/software/Rfam) [48] and the GenBank noncoding RNA database (http://www.ncbi.nlm.nih.gov/) were discarded. The retained 15–26 nt reads were mapped onto the the genome and ESTs of *Brassica napus*, *Brassica rapa* and *Brassica oleracea* using MapMi software under default parameters*.* Sequences with up to two mismatches were retained for miRNA prediction. After rigorous screening, all retained sequences of 15–26 nt with three or more copies in frequency were considered as potential miRNAs. We then attempted to align the predicted miRNAs to all rape known mature miRNA sequences in miRBase Version 17.0 [48] to identify novelty. Finally, Secondary structure prediction of individual miRNA was performed by MFOLD software (Version 2.38, http://mfold.rna.albany.edu/?q=mfold/RNA-Folding-Form) using the default folding conditions. [69].

The degradome analysis and the classification of target categories were performed using CleaveLand 2.0 [60]. Small RNA targets prediction was run against the transcriptome of interest. The alignment scores (using the [70] rubric) for each hit up to a user-defined cutoff were calculated, full RNA-RNA alignments were printed, and the 'cleavage site' associated with each prediction was also calculated. The cleavage site is simply the 10th nt of complementarity to the aligned small RNA. For randomized queries, no alignments were retained. However, concise records of each predicted target for the random queries were retained, including the predicted cleavage sites.

### End-point and SYBR Green I real-time PCR assays of *B. napus* miRNAs

End-point and Real-time looped RT-PCR [57] were used to validate and measure the levels of *B. napus* miRNA. Stem–loop RT primers, universal reverse primer and miRNA-specific forward primers for Bna-miR159, Bna-miR159b, Bna-miR160a, Bna-miR162a, Bna-miR165a, Bna-miR166e, Bna-miR167f, Bna-miR169a, Bna-miR171a*, Bna-miR390d, Bna-miR400, Bna-miR1140b, Bna-miRC2, Bna-miRC5-1, Bna-miRC5-6, Bna-miRC9, Bna-miRC17a-1, Bna-miRC18, Bna-miRC21, Bna-miRC22a-1, Bna-miRC30and Bna-miRC45 were designed according to Varkonyi-Gasic et al.[57]. (Additional file 4: Table S1). 1 μg of total RNA was reverse-transcribed to cDNA using ReverTra Ace (TOYOBO, Osaka, Japan). Stem-loop pulsed reverse transcription and end-point PCR was performed according to Varkonyi-Gasic et al. [57]. Advantage 2 PCR Polymerase Mix (Clontech, Mountain View, CA, USA) was used to perform end-point PCR. qRT-PCR was performed using SYBR Premix Ex Taq^TM^ of TaKaRa (TaKaRa Code: DRR041A) on an Applied Biosystems 7500 thermocycler (Applied Biosystems, Foster City, CA, USA). All reactions were run in triplicate. After the reaction, the threshold cycle (Ct) was determined using default threshold settings. The Ct is defined as the fractional cycle number at which the fluorescence passes the fixed threshold.

### Modified 5′ RNA ligase-mediated RACE for the mapping of mRNA cleavage sites

Total RNA from different tissues was extracted using Trizol (Invitrogen). Poly(A) + mRNA was purified from all kinds of pooled tissue RNA using the PolyA kit (Promega, Madison, WI), according to manufacturer’s instructions. A small RNA adapter (5′GUUCAGAGUUCUACAGUCCGACGAUC- 3) was ligated to Poly(A) + mRNA. A modified procedure for 5′ RNA ligase-mediated RACE (RLM-5 RACE) was followed with the 5-Full RACE Kit (TaKaRa, Dalian), according to manufacturer’s instructions. Nested PCR amplifications were performed using the 5 small RNA nested primer (5 AATGATACGGCGACCACCGACAGGTTCAGAGTTCTACAGTCCGA 3) and gene-specific nested primers (Additional file 8: Table S4). The amplification products were gel purified, cloned, and sequenced, and at least 10 independent clones were sequenced.

## Competing interests

The authors declare that they have no competing interests.

## Authors’ contributions

MYX and LW designed the study. MYX and YD performed experiments, analysed data and drafted the manuscript. QXZ and JS assisted with bioinformatic analysis and interpreting analysis results. YZL and LZ performed RLM-RACE experiment. YLF supervised the project and edited the manuscript. All authors discussed the results and implications and commented on the manuscript at all stages. All authors read and approved the final manuscript.

## Supplementary Material

Additional file 1: Figure S1 Secondary structures of 41 conserved *B.napus* miRNAs and miRNAs*. Pink and red section represents miRNA; yellow section represents miRNA*. Click here for file

Additional file 2: Table S2 Known miRNAs in *B. napus*. Click here for file

Additional file 3: Table S3 Four conserved miRNAs in *B. napus*. Click here for file

Additional file 4: Table S1 miRNA and primer sequences. Click here for file

Additional file 5: Figure S2 Secondary structures of 62 putative novel *B.napus* miRNAs and miRNAs*. Click here for file

Additional file 6: Figure S3 T-plots for targets of known miRNAs. Only one T-plot for target of one miRNA with same category was made. Click here for file

Additional file 7: Figure S4 T-plots for targets of brassica-specific miRNAs. Only one T-plot for target of one miRNA with same category was made. Click here for file

Additional file 8: Table S4 5RACE primer sequences. Click here for file
