# Supplementary material for: Identification of miRNAs and their targets from Brassica napus by high-throughput sequencing and degradome analysis
Source: BMC Genomics. 2012 Aug 24;13:421. doi: 10.1186/1471-2164-13-421 (PMC3599582; doi:10.1186/1471-2164-13-421)
Supplement: Additional file 1: Figure S1 — Secondary structures of 41 conserved B.napus miRNAs and miRNAs*. Pink and red section represents miRNA; yellow section represents miRNA*. [file 1471-2164-13-421-S1.pdf]

Figure S1. Secondary structures of 41 conserved *B.napus* miRNAs and miRNAs*.

Bna-miR159* Bna-miR159b Bna-miR160a/a* Bna-miR161* Bna-miR162b/b* Bna-miR165a

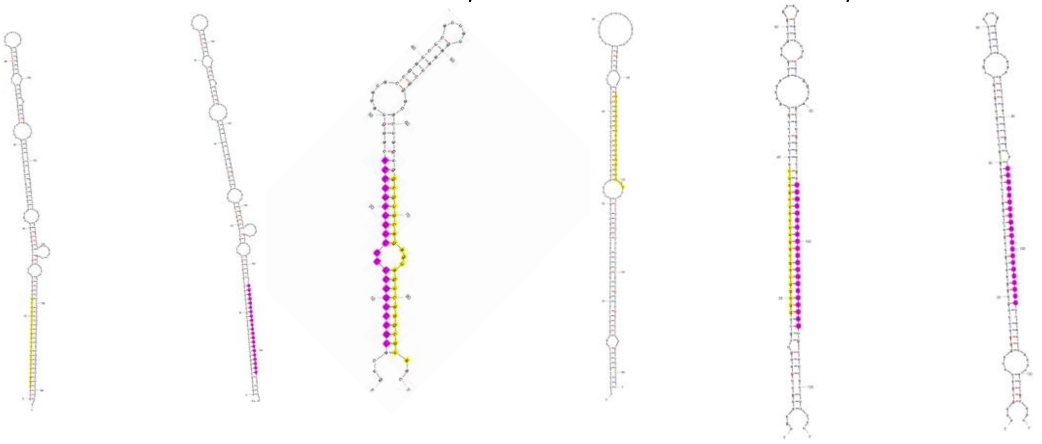

Bna-miR166a* Bna-miR166e Bna-miR166f Bna-miR167d Bna-miR167e/e* Bna-miR167f

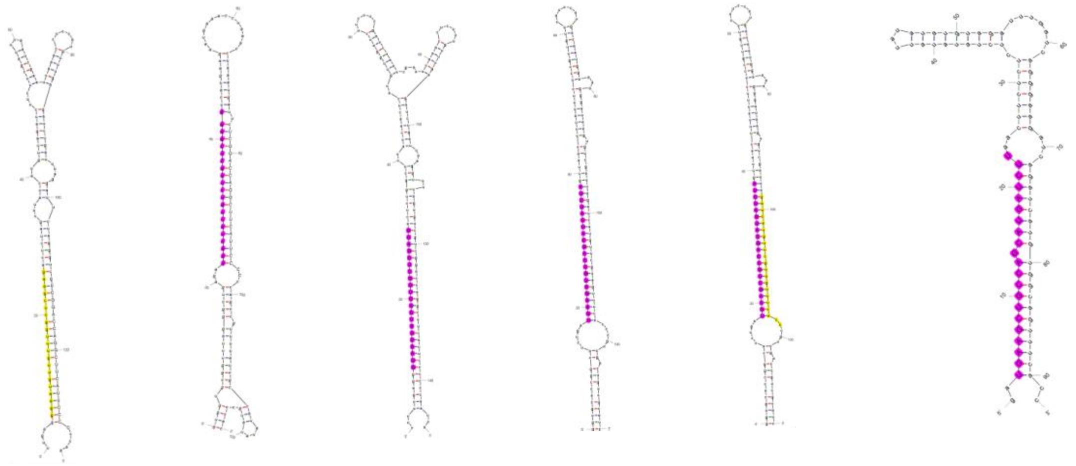

Bna-miR168b Bna-miR169n/n* Bna-miR171a* Bna-miR171f* Bna-miR162a Bna-miR172a/a*

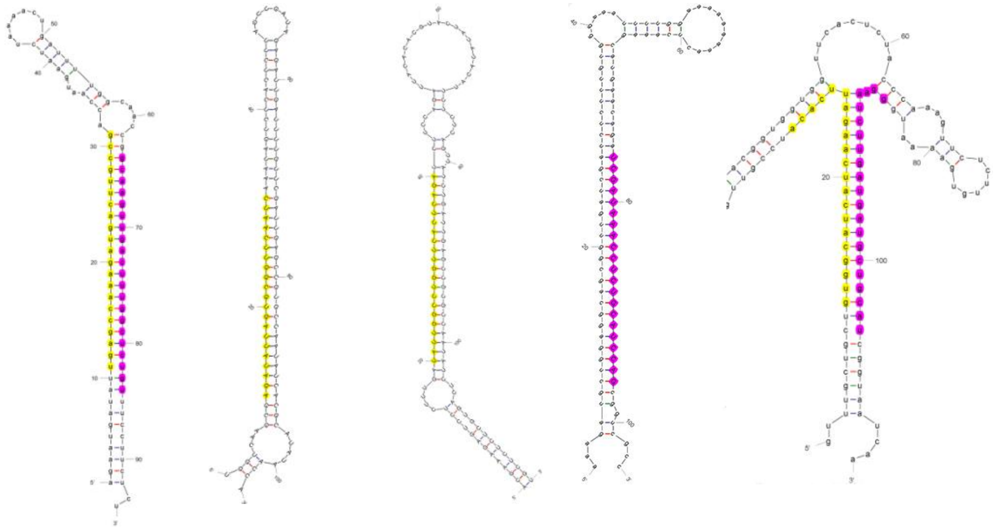

Bna-miR172b Bna-miR319a Bna-miR390d/d* Bna-miR393* Bna-miR396 Bna-miR398

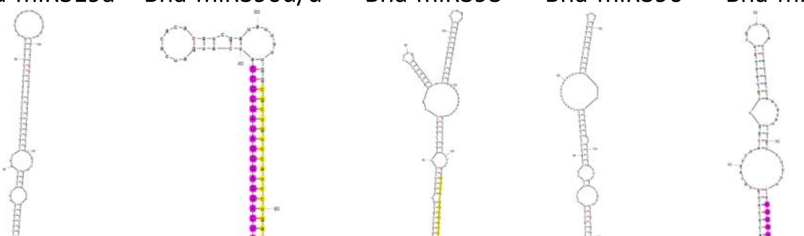

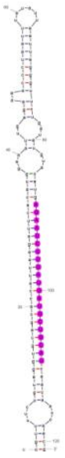

Bna-miR399d Bna-miR399f Bna-miR400 Bna-miR408a/a* Bna-miR2111c Bna-miR1140b/b*

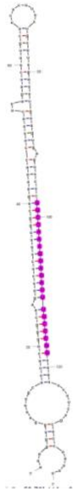
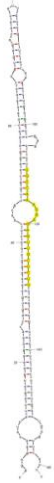
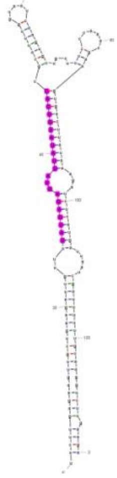
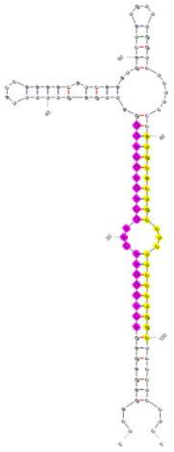
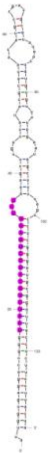
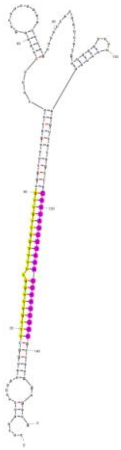

Bna-miR319b-1/b-2 Bna-miR824*

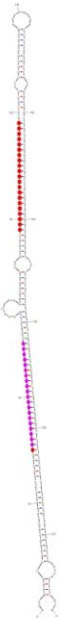
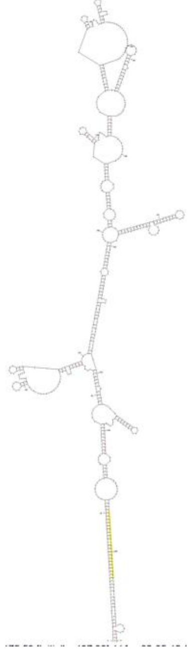

Pink and red represents miRNA; yellow represents miRNA*
