# Supplementary material for: Identification of miRNAs and their targets from Brassica napus by high-throughput sequencing and degradome analysis
Source: BMC Genomics. 2012 Aug 24;13:421. doi: 10.1186/1471-2164-13-421 (PMC3599582; doi:10.1186/1471-2164-13-421)
Supplement: Additional file 2: Table S2 — Known miRNAs in B. napus. [file 1471-2164-13-421-S2.pdf]

Table S2 Known miRNAs in *B. napus*

miRNA family	reads in *B. napus*	References
Bna-miR156b	63	Wang et al., 2007
Bna-miR159	16445	Wang et al., 2007
Bna-miR161	34	Wang et al., 2007
Bna-miR164	945	Wang et al., 2007
Bna-miR166a	6570	Wang et al., 2007
Bna-miR168	208	Wang et al., 2007
Bna-miR169a	8	Wang et al., 2007
Bna-miR169c	4	Wang et al., 2007
Bna-miR171a	30	Wang et al., 2007
Bna-miR171f	291	Wang et al., 2007
Bna -miR393	48	Xie et al., 2007
Bna-miR397a	19	Wang et al., 2007
Bna-miR824	41	Kutter et al., 2007
Bna-miR2111b*	25	Pant et al., 2009
