# Supplementary material for: Identification of miRNAs and their targets from Brassica napus by high-throughput sequencing and degradome analysis
Source: BMC Genomics. 2012 Aug 24;13:421. doi: 10.1186/1471-2164-13-421 (PMC3599582; doi:10.1186/1471-2164-13-421)
Supplement: Additional file 3: Table S3 — Four conserved miRNAs in B. napus. [file 1471-2164-13-421-S3.pdf]

Table S3 Four conserved miRNAs in *B. napus*.

miRNA	miR sequence (5' ⟶ 3')	miR length (nt)	Copy	genome	strand	Genomic position		Genomic position	
						miR start	miR end	precursor start	precursor end
Bna--miR166f	GGACTGTTGTCTGGCTCGAGG	21	50	AC189313	-	128843	128863	128758	128885
Bna-miR824*	CCTTCTCATCGATGGTCTAGA	21	14	AC189548	+	19239	19259	18652	19283
Bna –miR1140b	CAACAGCCTAAACCAATCGGA	21	25	AC189528	-	2998	3018	2986	3135
Bna –miR1140b*	TCCGATTGGCTTTAGGCTGTTG	22	13	AC189528	-	3096	3117	2986	3135
