# Supplementary material for: Identification of miRNAs and their targets from Brassica napus by high-throughput sequencing and degradome analysis
Source: BMC Genomics. 2012 Aug 24;13:421. doi: 10.1186/1471-2164-13-421 (PMC3599582; doi:10.1186/1471-2164-13-421)
Supplement: Additional file 4: Table S1 — miRNA and primer sequences. [file 1471-2164-13-421-S4.pdf]

Table S1 miRNA and primer sequences.

Bna-miR159	miRNA sequence<br>RT primer<br>Forward primer	TTTGGATTGAAGGGAGCTCTA<br>gtcgtatccagtgcagggtccgaggtattcgcactggatacgacTAGAGC<br>CGGCGGTTTGGATTGAAGGG
Bna--miR159b	miRNA sequence<br>RT primer<br>Forward primer	TTTGGATTGAAGGGAGCTCTT<br>gtcgtatccagtgcagggtccgaggtattcgcactggatacgacAAGAGC<br>GCGCGCTTTGGATTGAAGGG
Bna-miR160a	miRNA sequence<br>RT primer<br>Forward primer	TGCCTGGCTCCCTGTATGCCA<br>gtcgtatccagtgcagggtccgaggtattcgcactggatacgacTGGCAT<br>AGATCATGCCTGGCTCCCTG
Bna-miR162a	miRNA sequence<br>RT primer<br>Forward primer	TCGATAAACCTCTGCATCCAG<br>gtcgtatccagtgcagggtccgaggtattcgcactggatacgacCTGGAT<br>GCGCGCTCGATAAACCTCTG
Bna-miR165a	miRNA sequence<br>RT primer<br>Forward primer	TCGGACCAGGCTTCATCCCCC<br>gtcgtatccagtgcagggtccgaggtattcgcactggatacgacGGGGGA<br>ACTGCATCGGACCAGGCTTC
Bna-miR166e	miRNA sequence<br>RT primer<br>Forward primer	GGAATGTTGTCTGGCTCGAGG<br>gtcgtatccagtgcagggtccgaggtattcgcactggatacgacCCTCGA<br>GCGCTCGGAATGTTGTCTGG
Bna-miR167f	miRNA sequence<br>RT primer<br>Forward primer	TGAAGCTGCCAGCATGATCTT<br>gtcgtatccagtgcagggtccgaggtattcgcactggatacgacTAGATC<br>ATGCGCATGAAGCTGCCAGC
Bna-miR169a	miRNA sequence<br>RT primer<br>Forward primer	CAGCCAAGGATGACTTGCCGA<br>gtcgtatccagtgcagggtccgaggtattcgcactggatacgacTCGGCA<br>CGAGTCCAGCCAAGGATGAC
Bna-miR171a*	miRNA sequence<br>RT primer<br>Forward primer	TTGAGCCGTGCCAATATCACT<br>gtcgtatccagtgcagggtccgaggtattcgcactggatacgacAGTGAT<br>GTTAACTTGAGCCGTGCC
Bna -miR390d	miRNA sequence<br>RT primer<br>Forward primer	AAGCTCAGGAGGGATAGCGCC<br>gtcgtatccagtgcagggtccgaggtattcgcactggatacgacGGCGCT<br>GTCTCGTAAGCTCAGGAGGG
Bna-miR400	miRNA sequence<br>RT primer<br>Forward primer	TATGAGAGTATTATAAGTCAC<br>gtcgtatccagtgcagggtccgaggtattcgcactggatacgacGTGACT<br>GCGCGGTATGAGAGTATT
Bna –miR1140b	miRNA sequence<br>RT primer<br>Forward primer	CAACAGCCTAAACCAATCGGA<br>gtcgtatccagtgcagggtccgaggtattcgcactggatacgacTCCGAT<br>GACCCAACAGCCTAAACC
Bna-miRC2	miRNA sequence<br>RT primer<br>Forward primer	ATAAATCCCAAGCATCATCCA<br>gtcgtatccagtgcagggtccgaggtattcgcactggatacgacTGGATG<br>GCGCCATAAATCCCAAGC
Bna-miRC5-1	miRNA sequence<br>RT primer<br>Forward primer	TGTGTTGTGATGATAATCCGA<br>gtcgtatccagtgcagggtccgaggtattcgcactggatacgacTCGGAT<br>GCGCCGTGTGTTGTGATGAT

Bna-miRC5-6	miRNA sequence<br>RT primer<br>Forward primer	TCGGATTATCATCACAACACT<br>gtcgtatccagtgcagggtccgaggtattcgcactggatacgacAGTGTT<br>GCGCGTCGGATTATCATCAC
Bna-miRC9	miRNA sequence<br>RT primer<br>Forward primer	TGCCTGGCTCCCTGTATACCA<br>gtcgtatccagtgcagggtccgaggtattcgcactggatacgacTGGTAT<br>TAATTGCCTGGCTCCCTG
Bna-miRC17a-1	miRNA sequence<br>RT primer<br>Forward primer	TTTCCAAATGTAGACAAAGCA<br>gtcgtatccagtgcagggtccgaggtattcgcactggatacgacTGCTTT<br>GCGCGTTTCCAAATGTAG
Bna-miRC18	miRNA sequence<br>RT primer<br>Forward primer	TCGCGATCTTAGATCCTCTAA<br>gtcgtatccagtgcagggtccgaggtattcgcactggatacgacTTAGAG<br>GCCTCGCGATCTTAGATC
Bna-miRC21	miRNA sequence<br>RT primer<br>Forward primer	GGAGGCAGCGGTTGATCGATC<br>gtcgtatccagtgcagggtccgaggtattcgcactggatacgacGATCGA<br>TATAGGAGGCAGCGGTTG
Bna-miRC22a-1	miRNA sequence<br>RT primer<br>Forward primer	CAAGTAGACGACTTTCCAGAC<br>gtcgtatccagtgcagggtccgaggtattcgcactggatacgacGTCTGG<br>GCGCCAAGTAGACGACTT
Bna-miRC30	miRNA sequence<br>RT primer<br>Forward primer	TCCTGGACGACTTTCAAGTAAG<br>gtcgtatccagtgcagggtccgaggtattcgcactggatacgacCTTACT<br>GCATCCTGGACGACTTTC
Bna-miRC45	miRNA sequence<br>RT primer<br>Forward primer	TTTCATCTTAGAGAATGTTGTC<br>gtcgtatccagtgcagggtccgaggtattcgcactggatacgacGACAAC<br>GCGCGCGTTTCATCTTAGAG
Universal RV	Reverse primer	GTGCAGGGTCCGAGGTATTC
