# Supplementary material for: Identification of miRNAs and their targets from Brassica napus by high-throughput sequencing and degradome analysis
Source: BMC Genomics. 2012 Aug 24;13:421. doi: 10.1186/1471-2164-13-421 (PMC3599582; doi:10.1186/1471-2164-13-421)

Figure S2. Secondary structures of 62 putative novel *B.napus* miRNAs and miRNAs*.

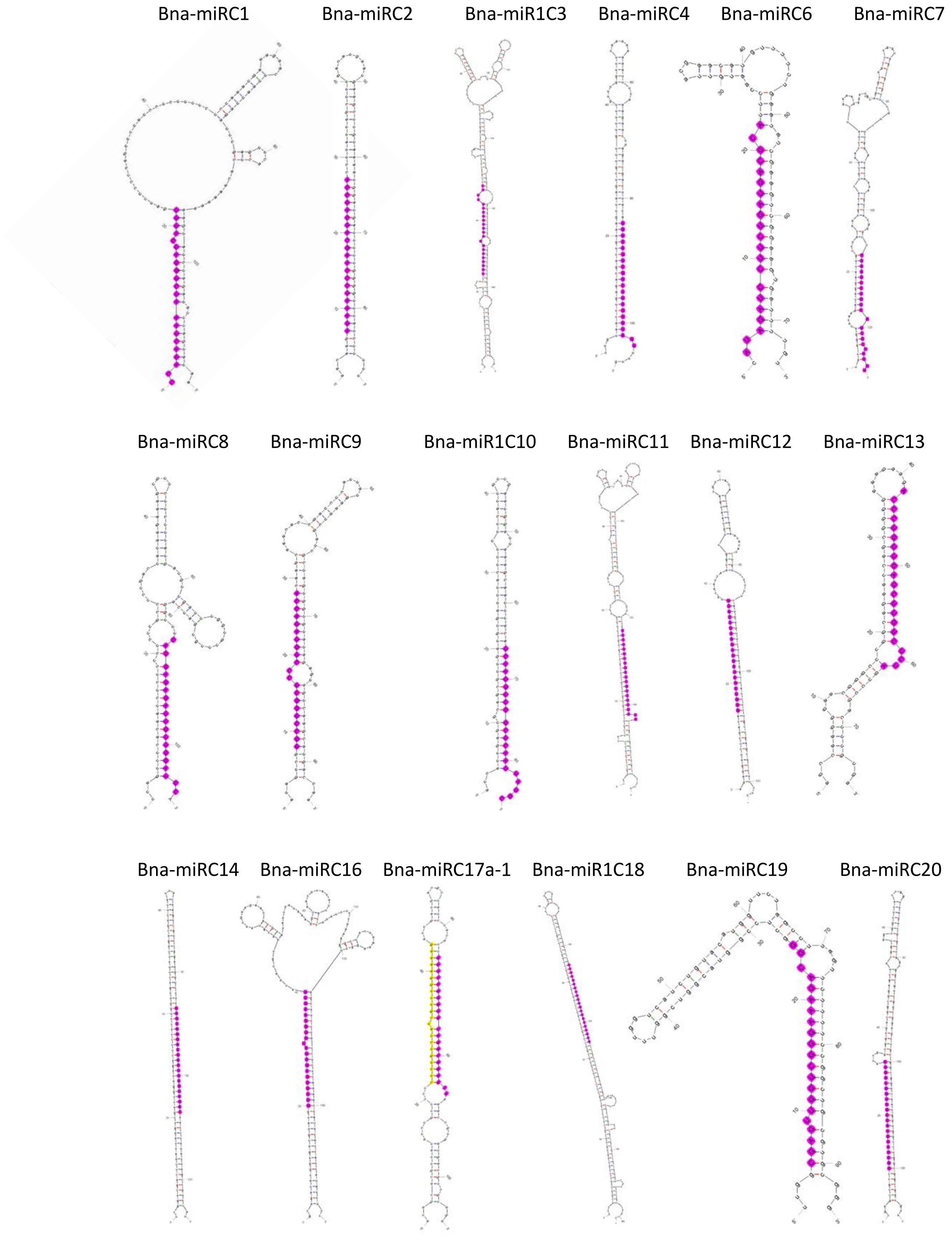

Bna-miRC21 Bna-miRC24 Bna-miRC25/25* Bna-miR1C26 Bna-miRC27

Bna-miRC28

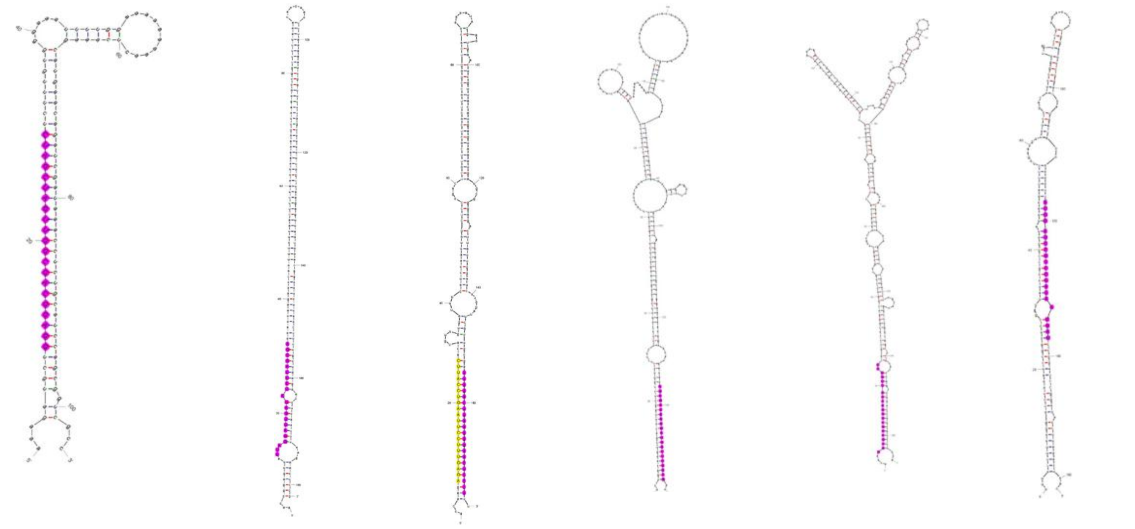

Bna-miRC29 Bna-miRC30 Bna-miRC31 Bna-miR1C32 Bna-miRC33 Bna-miRC34

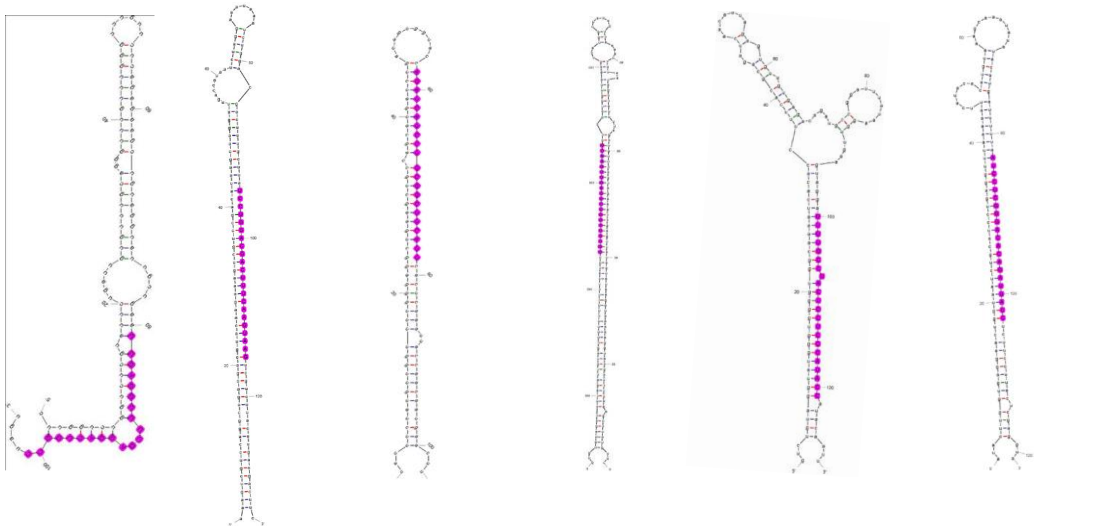

Bna-miRC35 Bna-miRC36 Bna-miRC37 Bna-miRC38 Bna-miR1C39 Bna-miRC40

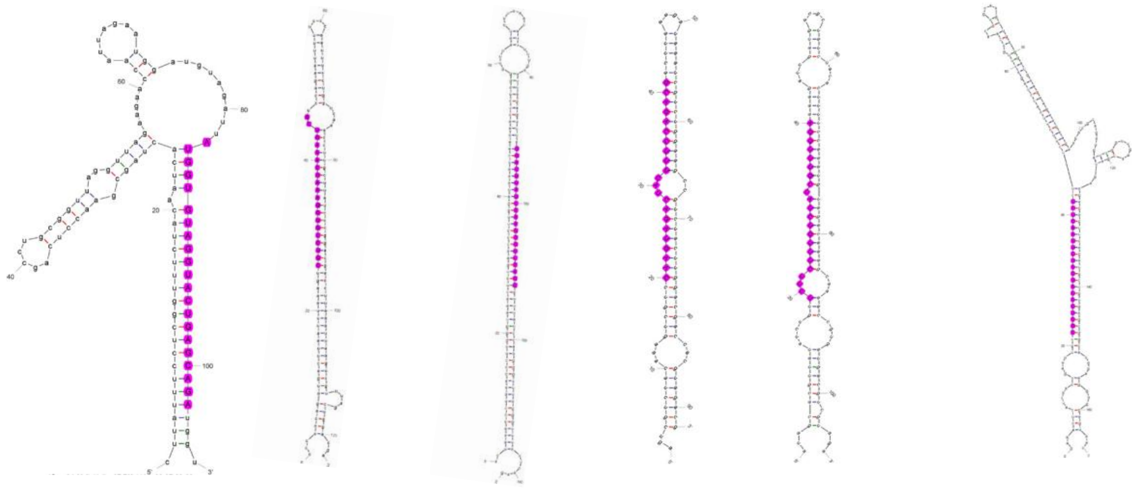

Bna-miRC41 Bna-miRC42 Bna-miRC43 Bna-miR1C44 Bna-miRC45 Bna-miRC46 Bna-miRC47

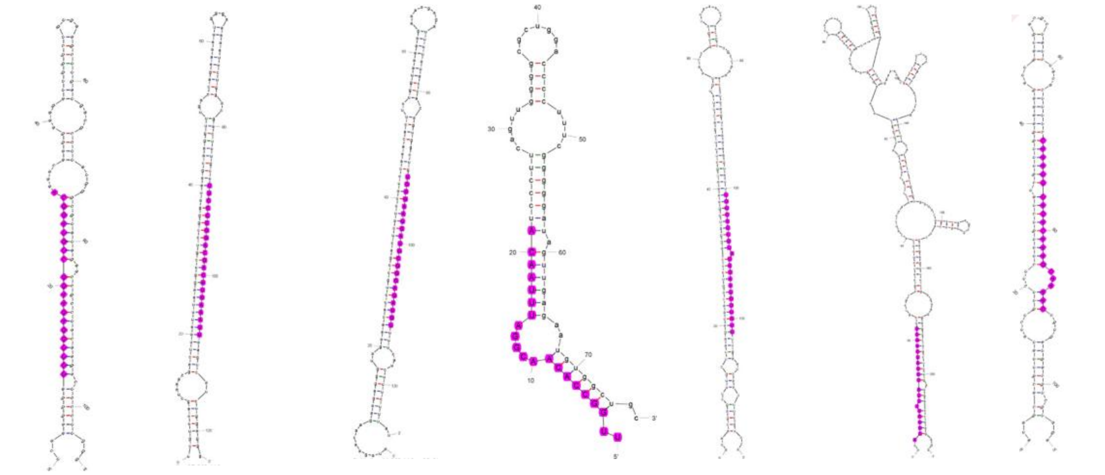

Pink represents miRNA; yellow represents miRNA*

Bna-miRC5 family

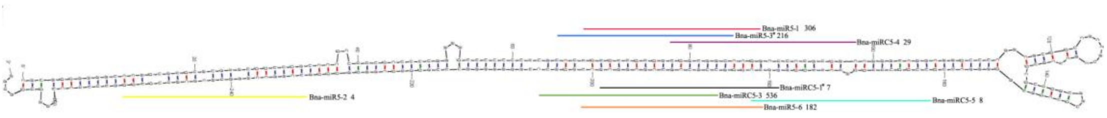

Bna-miRC15 family

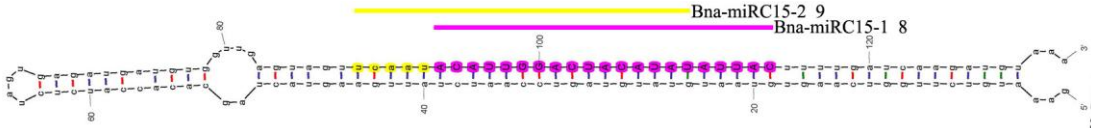

Bna-miRC22a family

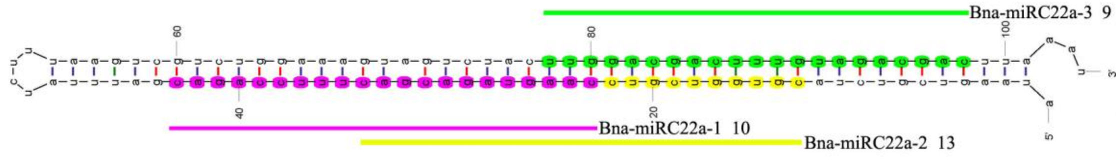

Bna-miRC23a family

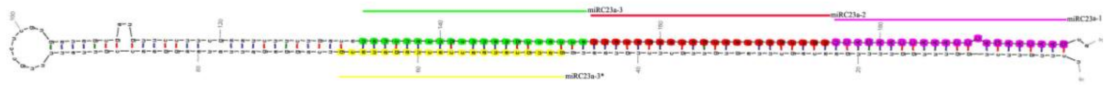

Supplement: Additional file 5: Figure S2 — Secondary structures of 62 putative novel B.napus miRNAs and miRNAs*. [file 1471-2164-13-421-S5.pdf]
