# Supplementary material for: Identification of miRNAs and their targets from Brassica napus by high-throughput sequencing and degradome analysis
Source: BMC Genomics. 2012 Aug 24;13:421. doi: 10.1186/1471-2164-13-421 (PMC3599582; doi:10.1186/1471-2164-13-421)
Supplement: Additional file 6: Figure S3 — T-plots for targets of known miRNAs. Only one T-plot for target of one miRNA with same category was made. [file 1471-2164-13-421-S6.pdf]

Bna-miR156a （EL625881）

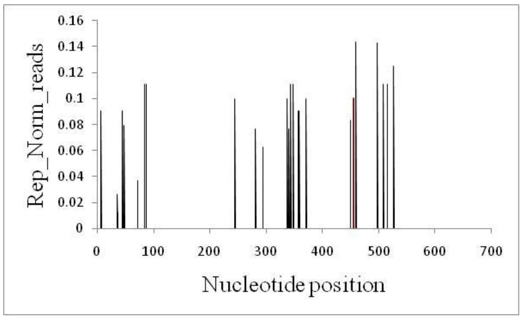

Bna-miR164b （ES906443）

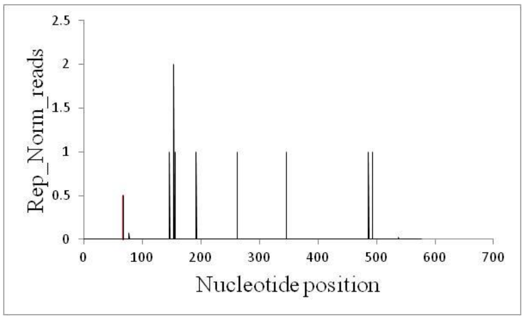

Bna-miR169a (EE543166)

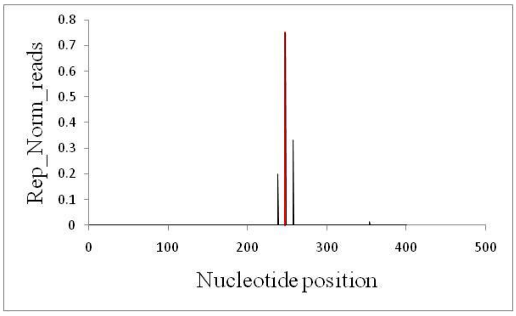

Bna-miR169a (CN729971)

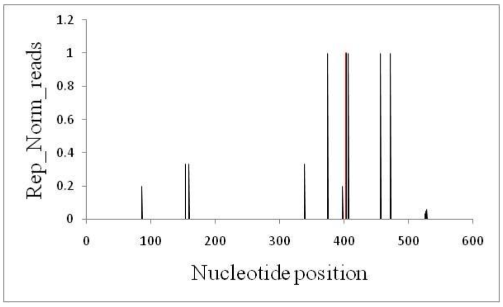

Bna-miR169e (ES980547)

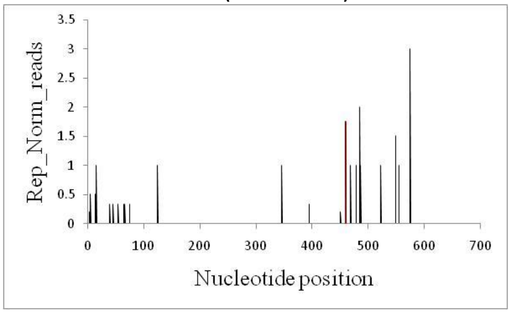

Bna-miR169e (EE543166)

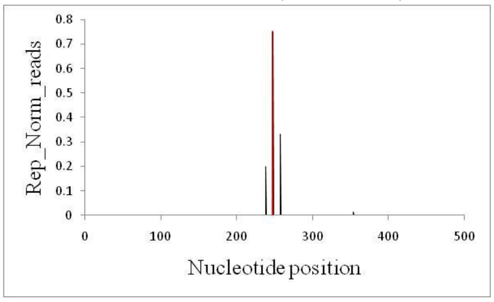

Bna-miR169e (CN729971)

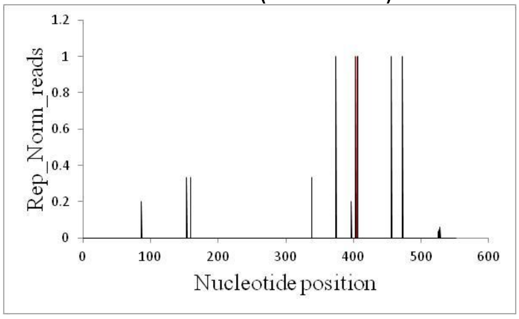

Bna-miR172f (FG568924)

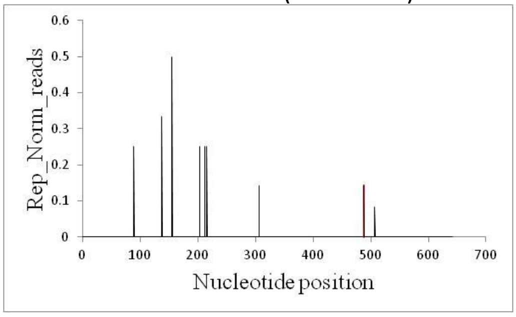

Bna-miR172f (EV197066)

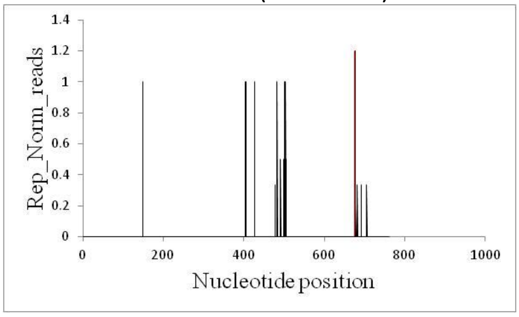

Bna-miR172f (ES967403)

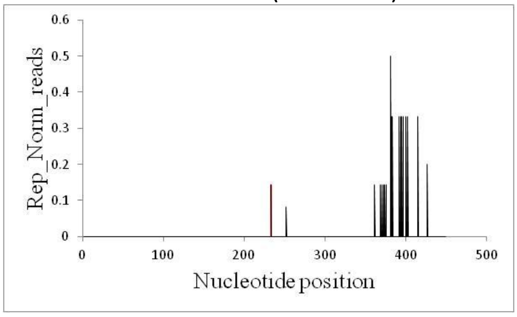

Bna-miR172f (DY025256)

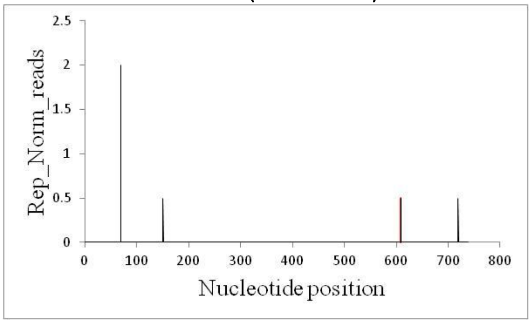

Bna-miR390a/d (EV220086)

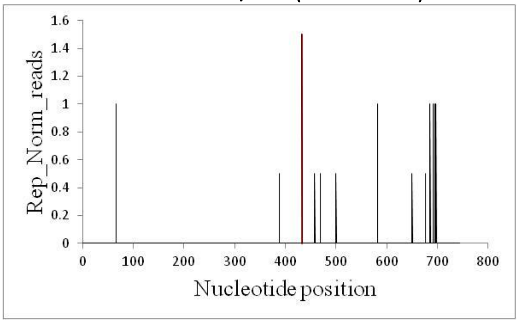

Bna-miR393 (EL628991)

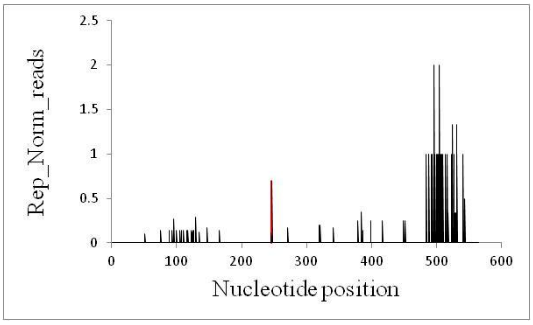

Bna-miR393 (EV176346)

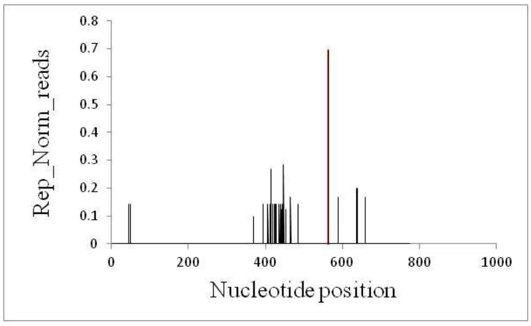

Bna-miR396 (EL628991)

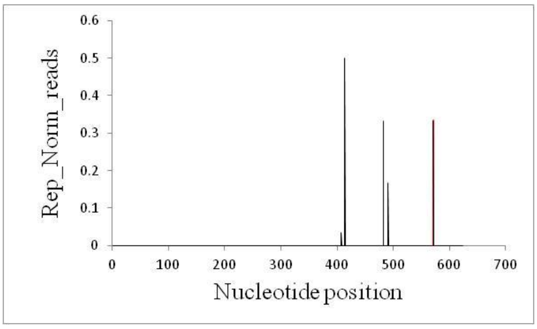

Bna-miR408a (EE417826)

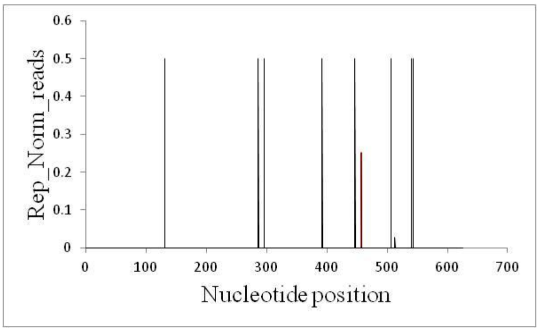

Bna-miR408b (EV075738)

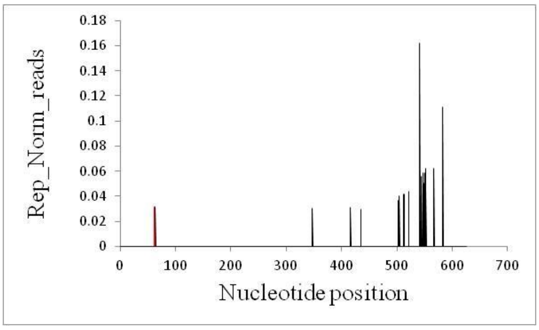

Bna-miR824 (EV112524)

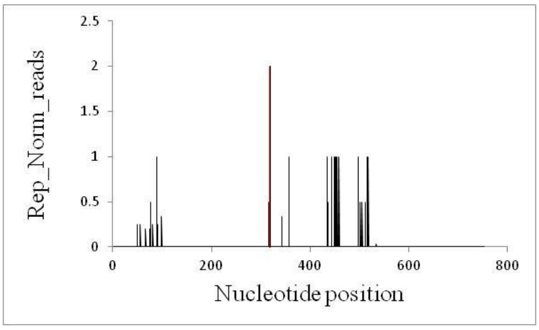

Bna-miR1140b (EV217683)

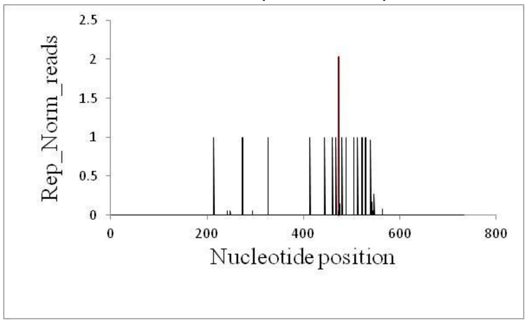

Bna-miR1140b (ES912747)

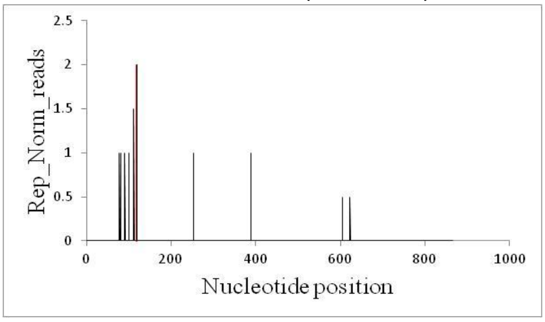

Bna-miR2111b (EV221566)

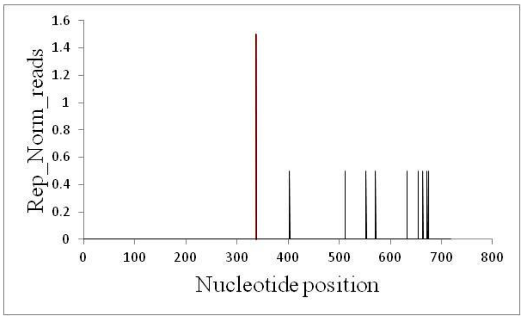

Figure S3. T-plots for targets of known miRNAs.

We used normalized numbers in plotting the cleavages on target mRNAs, which were referred to as ‘target plots’ (t-plots) by German et al [51]. Signature abundance throughout the length of the indicated transcripts is shown. Representative t-plots for class 0 (a), class I (b), class II (c), class III (d), and class VI (e) categories are shown. Arrows indicate signatures consistent with miRNA-directed cleavage. miRNA:mRNA alignments along with the detected cleavage frequencies (normalized numbers) are shown. The frequencies of degradome tags with 5′ends at the indicated positions are shown in black, with the frequency at position 10 of the inset miRNA target alignment highlighted in red. The underlined nucleotide on the target transcript indicates the cleavage site detected in the degradome.
