# Supplementary material for: Identification of miRNAs and their targets from Brassica napus by high-throughput sequencing and degradome analysis
Source: BMC Genomics. 2012 Aug 24;13:421. doi: 10.1186/1471-2164-13-421 (PMC3599582; doi:10.1186/1471-2164-13-421)
Supplement: Additional file 8: Table S4 — 5RACE primer sequences. [file 1471-2164-13-421-S8.pdf]

Table S4 5′RACE primer sequences

miRNAs	Target EST	GSPO	GSPI
Bna-miR160a	EV006535	TAGTGGTGTTGCTGTTAGGCG	TGTTAGGCGACAGCTCATCC
Bna-miR156a	EL625881	CCTTCAAGCATTTACTTGGG	GCAGATAGCTAGAGATTAC
Bna-miRC2	EV142354	ATCTGCAGAGAAGCCACACC	GTCCAATCCACCATCCACTAAC
Bna-miRC18	GT074945	CCTGACCAGTCTTGAACTCAG	GAACTCAGCCAAGTACCTGTG
Bna-miRC22a-1	EV044066	CTCCGTTCCAGAAATGTCTTG	CGCGGCGTCTTGAAAATTAAAC
RACE 5′primer	AATGATACGGCGACCACCGACAGGTTCAGAGTTCTACAGTCCGA		
